# The Chromatin Assembly Factor 1 Promotes Rad51-Dependent Template Switches at Replication Forks by Counteracting D-Loop Disassembly by the RecQ-Type Helicase Rqh1

**DOI:** 10.1371/journal.pbio.1001968

**Published:** 2014-10-14

**Authors:** Violena Pietrobon, Karine Fréon, Julien Hardy, Audrey Costes, Ismail Iraqui, Françoise Ochsenbein, Sarah A.E. Lambert

**Affiliations:** 1 Institut Curie, Centre de Recherche, Orsay, France; 2 Centre national de la Recherche Scientifique, UMR3348, Centre Universitaire, Orsay, France; 3 Commissariat à l'Energie Atomique, iBiTec-S, Service de Biologie Intégrative et de Génétique Moléculaire, Gif-sur-Yvette, France; National Cancer Institute, United States of America

## Abstract

At blocked replication forks, homologous recombination mediates the nascent strands to switch template in order to ensure replication restart, but faulty template switches underlie genome rearrangements in cancer cells and genomic disorders. Recombination occurs within DNA packaged into chromatin that must first be relaxed and then restored when recombination is completed. The chromatin assembly factor 1, CAF-1, is a histone H3-H4 chaperone involved in DNA synthesis-coupled chromatin assembly during DNA replication and DNA repair. We reveal a novel chromatin factor-dependent step during replication-coupled DNA repair: Fission yeast CAF-1 promotes Rad51-dependent template switches at replication forks, independently of the postreplication repair pathway. We used a physical assay that allows the analysis of the individual steps of template switch, from the recruitment of recombination factors to the formation of joint molecules, combined with a quantitative measure of the resulting rearrangements. We reveal functional and physical interplays between CAF-1 and the RecQ-helicase Rqh1, the BLM homologue, mutations in which cause Bloom's syndrome, a human disease associating genome instability with cancer predisposition. We establish that CAF-1 promotes template switch by counteracting D-loop disassembly by Rqh1. Consequently, the likelihood of faulty template switches is controlled by antagonistic activities of CAF-1 and Rqh1 in the stability of the D-loop. D-loop stabilization requires the ability of CAF-1 to interact with PCNA and is thus linked to the DNA synthesis step. We propose that CAF-1 plays a regulatory role during template switch by assembling chromatin on the D-loop and thereby impacting the resolution of the D-loop.

## Abstract 2

A molecular switch for times of replication stress - Chromatin Assembly Factor 1 helps to protect DNA during recombination-mediated template-switching, favoring the rescue of stalled replication forks by both beneficial and detrimental homologous recombination events.

## Author Summary

Obstacles to the progression of DNA replication forks can result in genome rearrangements that are often observed in cancer cells and genomic disorders. Homologous recombination is a mechanism of restarting stalled replication fork that involves synthesis of the new DNA strands switching templates to a second (allelic) copy of the DNA sequence. However, the new strands can also occasionally recombine with nonallelic repeats (distinct regions of the genome that resemble the correct one) and thereby cause the inappropriate fusion of normally distant DNA segments; this is known as faulty template switching. The chromatin assembly factor 1 (CAF-1) is already known to be involved in depositing nucleosomes on DNA during DNA replication and repair. We have found that CAF-1 is also involved in the recombination-mediated template switch pathway in response to replication stress. Using both genetic and physical assays that allow the different steps of template switch to be analyzed, we reveal that CAF-1 protects recombination intermediates from disassembly by the RecQ-type helicase Rqh1, the homologue of BLM (people with mutations that affect BLM have Bloom's syndrome, an inherited predisposition to genome instability and cancer). Consequently, the likelihood of faulty template switch is controlled by the antagonistic activities of CAF-1 and Rqh1. We thus identified an evolutionarily conserved interplay between CAF-1 and RecQ-type helicases that helps to maintain genome stability in the face of replication stress.

## Introduction

The maintenance of genome stability requires a complex network to coordinate multiple pathways, including DNA replication, repair, and recombination in a chromatin context. Replication stress, including obstacles to replication fork progression, has emerged as a major source of genome instability that fuels cancer development and underlies chromosome modifications observed in genomic disorders [1]. Deciphering the control of repair pathways occurring at replication forks remains of crucial importance to understanding of the mechanisms underlying genome rearrangements.

Homologous recombination (HR) is an evolutionarily conserved mechanism that promotes DNA repair and contributes to accurate and complete DNA replication [2]. When fork progression is disrupted by DNA damage or a fork obstacle, HR mediates the nascent strands to switch templates to resume DNA synthesis. Template switch occurs either at the three-way branched junction of the fork to restart it or between sister-chromatids to fill in single-stranded DNA (ssDNA) gaps left behind the moving fork [3],[4]. This last pathway is referred to as error-free postreplication repair (PRR) [5].

Faulty replication restart events are one of the causal mechanisms of genome instability. When control of allelic recombination fails, nascent strands at a blocked replication fork can recombine with a nonallelic homologous repeat and initiate DNA synthesis on a noncontiguous template, thus resulting in the fusion of noncontiguous DNA segments and genome rearrangements [6]–[8]. This mechanism is referred to as faulty template switch and is proposed to drive genome rearrangements in cancers cells (e.g., chromothripsis) and in genomic disorders (e.g., complex rearrangements such as triplication-associated inversions) [1],[9]. Faulty template switch between homologous repeats is reminiscent of nonallelic HR (NAHR). In yeast, inverted repeats are particularly prone to faulty template switching [10]. Recently, both HR and error-free PRR have been reported as mechanisms of faulty replication leading to fusion of inverted repeats in human cells [11]. Thus, the mechanisms of faulty template switch appear evolutionarily conserved.

HR has been extensively studied in the context of double-strand break (DSB) repair, but only a few studies have addressed the mechanisms of template switch [2],[4],[12]. With the assistance of HR mediators (such as Rad52 in yeast), the recombinase Rad51 nucleates onto ssDNA covered by RPA to form a nucleoprotein filament. After the search for homology, the Rad51 filament invades a homologous DNA duplex to pair the invading ssDNA with the complementary strand, whereas the noncomplementary strand is displaced. The resulted three-stranded intermediate is a type of joint molecule (JM) called a displacement loop (D-loop) in which the 3′ end of the invading strand primes DNA synthesis. At replication forks, extension of the D-loop by DNA synthesis might permit the restoration of a functional replisome, thus ensuring the completion of DNA replication [13]. In the context of DSB repair, the capture of the second DNA end results in the formation of a later JM called a double Holliday junction (dHJ) whose resolution by cleavage leads to crossover (CO) formation, a source of chromosome rearrangements associated with NAHR [1],[12],[14]. Several DNA helicases/translocases have been shown to be involved in the prevention of mitotic CO by preventing D-loop formation or its disassembly—among them, Srs2, FANCM, and RecQ-type helicases [15]–[23]. Whether the outcomes of template switch at replication forks are also regulated by helicase-dependent D-loop dismantling is unknown.

HR occurs within DNA packaged into chromatin that needs to be disassembled and then restored after the recombination event is completed [24]. Chromatin remodeling factors help in relaxing chromatin and in providing access to DNA damage signaling and repair machineries at damaged sites, but how chromatin restoration is coupled to HR remains poorly understood. The chromatin assembly factor 1, CAF-1, is a histone H3-H4 chaperone that promotes DNA synthesis-coupled chromatin assembly during DNA repair and DNA replication [25]–[28]. CAF-1 is a three-subunit complex conserved throughout evolution, and the three CAF-1 subunits in *Schizosaccharomyces pombe* are called Pcf1 (SPBC29A10.03c), Pcf2 (SPAC26H5.03), and Pcf3 (SPAC25H1.06), which correspond, respectively, to the p150, p60, and p48 in mammalian cells [29]. The large subunit of CAF-1, p150, interacts with PCNA, thus targeting CAF-1 to DNA synthesis sites at which CAF-1 and Asf1 (anti-silencing factor 1) cooperatively assemble chromatin onto newly synthesized DNA in a PCNA-dependent manner [30]–[37].

In response to DNA damage, the large subunit of CAF-1 and the heterochromatin factors HP1 (heterochromatin protein 1) are targeted to mammalian HR foci, within which they promote the resection of DSBs and thus the recruitment of HR factors such as Rad51 [38]–[41]. After completion of DNA repair, CAF-1 and Asf1 restore nucleosomal organization at DNA damage [26]–[28],[30],[42]–[44]. In budding yeast, CAF-1 and Asf1 are dispensable for DSB repair by HR but necessary for the restoration of the chromatin state, a step required to turn off checkpoint activation [45],[46]. It is suggested that CAF-1 primes DSB repair and then switches to an active histone chaperone mode to restore chromatin at DNA damage [24]. Whether CAF-1 regulates other HR pathways such as template switch and whether it impacts repair fidelity is unknown.

Here, we identified that fission yeast CAF-1 acts in a HR pathway alternative to PRR when cells replicate a damaged template. We revealed functional and physical interactions between CAF-1 and the RecQ-type helicase Rqh1, the fission yeast BLM homologue. Using a conditional replication fork obstacle, we report a novel chromatin factor-dependent step during HR-mediated template switch: CAF-1 counteracts the disassembly of D-loop intermediates by Rqh1. As a consequence, the likelihood of faulty template switch is controlled by the antagonistic roles of CAF-1 and Rqh1 in D-loop stability. The protection of the D-loop requires the three CAF-1 subunits and its ability to interact with PCNA, showing that CAF-1 stabilizes the D-loop at the DNA synthesis step. Thus, CAF-1 and Rqh1 act coordinately to maintain genome stability in response to replication stress. We propose that CAF-1 plays a regulatory role during template switch by assembling chromatin on the D-loop and thereby impacting its resolution.

## Results

### CAF-1 Acts in a Replication-Coupled DNA Repair Pathway Independently of the Error-Prone and Error-Free Branches of PRR

**Figure 1:**
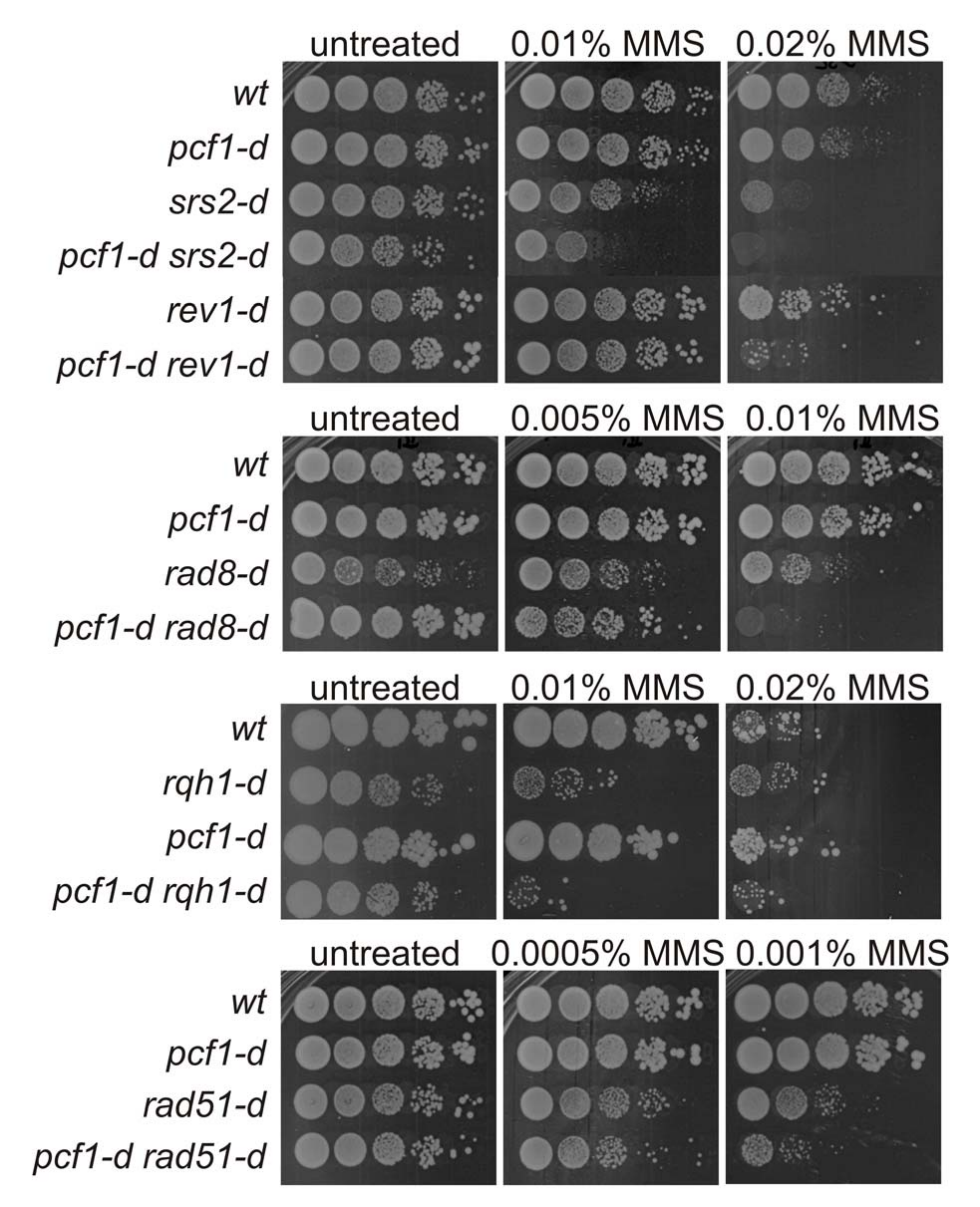
CAF-1 promotes replication-coupled DNA repair independently of the error-prone and error-free branch of PRR. Serial 10-fold dilution from indicated strains spotted onto media containing indicated MMS concentration.

We wanted to ascertain whether CAF-1 was involved in replication-coupled DNA repair. We focused on cell resistance to the alkylating agent methyl-methane sulfonate (MMS) that creates DNA lesions blocking fork elongation and known to induce template switch events [4]. The deletion of *pcf1* (*pcf1-d*, SPBC29A10.03c) did not affect cell sensitivity to MMS compared to wild-type (*wt*) cells. However, combined with genetic backgrounds in which error-prone PRR (the bypass of DNA lesions by translesion synthesis, *rev1-d*, SPBC1347.01c) or error-free PRR (*rad8-d*, SPAC13G6.01c, Rad8 being the homologue of budding yeast Rad5) were defective, *pcf1-d* resulted in an increased cell sensitivity to MMS, compared to each single mutant (Figure 1). A similar genetic interaction was observed with Srs2 (SPAC4H3.05), a helicase involved in error-free PRR [47]. These data suggest that CAF-1 acts in a replication-coupled DNA repair pathway but independently of the error-prone and error-free branches of PRR. We then asked whether CAF-1 could act in the Rad51 (SPAC644.14c)-dependent HR pathway. We found that the double mutant *pcf1-d rad51-d* exhibited only a modest increased sensitivity to MMS compared to the single mutant *rad51-d* (Figure 1). These data indicate that CAF-1 may operate in the Rad51-dependent replication-coupled DNA repair pathway but may not function entirely through the HR pathway.

### Conditional Replication Fork Obstacles to Investigate HR-Mediated Template Switch

To decipher the role of CAF-1 in replication-coupled HR, we made use of a polar replication fork barrier (RFB), which is genetically encoded by a DNA sequence called *RTS1* bound by the protein Rtf1 whose expression is regulated at the transcriptional level via the use of the *nmt41* promoter. In the presence of thiamine in the media, Rtf1 is not expressed and the *RTS1*-RFB is not active; after at least 16 h following thiamine removal, Rtf1 is expressed and the *RTS1*-RFB is induced [48]. In the *t-ura4 <ori* construct, a single *RTS1*-RFB is located near an efficient replication origin (ori 3006/7, on chromosome III) to allow the block of forks emanating from this origin and moving toward the telomere, the main replication direction of the *ura4* locus (Figure S1A). Blocked replication forks are restarted by HR and independently of DSBs [3],[7].

Perturbed replication-coupled chromatin assembly, due to CAF-1 and Asf1 deficiency, leads to a higher susceptibility of replication forks to collapse and thus an increased level of genome instability in budding yeast [49]–[52]. We thus asked whether a defect in CAF-1 affects the activity of the *RTS1*-RFB and the early steps of HR at replication forks. At the *t-ura4 <ori* locus, which contains a single fork barrier, the analysis of replication intermediates (RIs) by bidimensional gel electrophoresis (2DGE) showed that the *RTS1*-RFB was as efficient in the absence of CAF-1 (i.e., in either *pcf1-d*, *pcf2-d*, or *pcf3-d* null mutant) as in the *wt* strain (Figure S1B–C). Also, Rad52, the main HR factor (SPAC30D11.10), was recruited to the fork barrier in the absence of CAF-1 to the same extent as in the *wt* strain (Figure S1D). Our data indicate that the *RTS1*-RFB was functional and prone to recruit HR factors in the absence of CAF-1.

**Figure 2:**
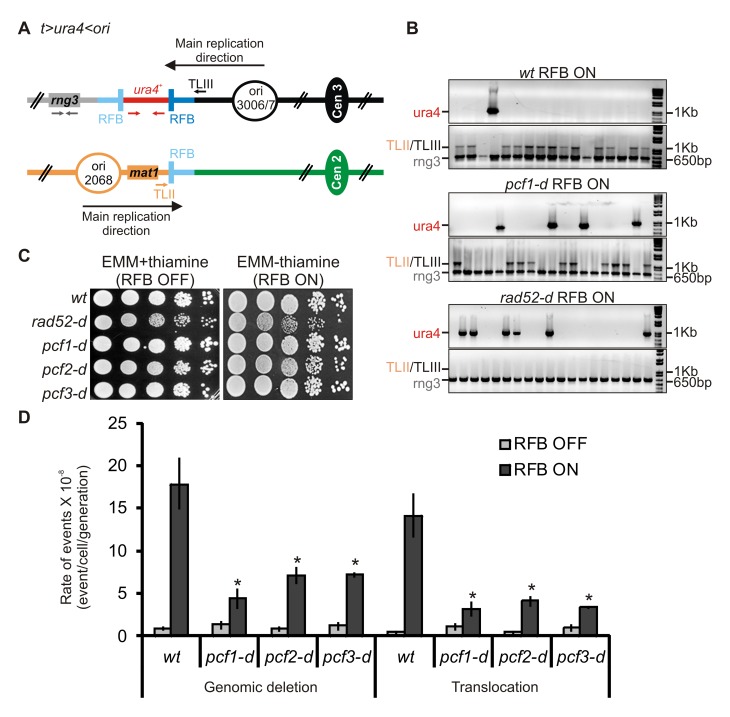
CAF-1 promotes faulty template switch at blocked replication forks. (A) Diagram of the *t> ura4 <ori* locus, in which *t* refers to the telomere (gray lines), *ura4* refers to the *wt* gene (red lines),> *and* <refers to the polarity of the two *RTS1*-RFB (blue bars, the darkest blue one corresponds to the most efficient RFB), and *ori* refers to the replication origin (opened black circle) on the centromere-proximal side. A *RTS1*-RFB is naturally located on chromosome II. The green and black circles indicate the centromere of chromosomes II and III, respectively. Fork arrest at the *RTS1*-RFB on chromosome III leads to ectopic recombination with the *RTS1* sequence located on chromosome II, resulting in *ura4* loss and genomic deletion associated or not with a translocation between chromosomes II and III. The loss of *ura4* is genetically selected using the 5-FOA drug that allows the selection of cells exhibiting a loss of *ura4^+^* function (deletion or mutation). Primers used for amplifying the 1 Kb *ura4* fragment or the 650 bp *rng3* fragment are depicted in red and grey, respectively. Primers used to amplify the translocation junction (1.2 kb) are represented in orange on chromosome II (TLII) and in black on chromosome III (TLIII). (B) Representative PCR-amplification from 5-FOA–resistant colonies from indicated strains and conditions. PCR products and their sizes are indicated on the figure. (C) Survival of indicated strains upon fork arrest at the *t> ura4 <ori* locus. Serial 10-fold dilution from indicated strains spotted onto media containing thiamine (RFB OFF) or not (RFB ON). (D) Rate of genomic deletion and translocation for the strains indicated; ON and OFF refers to the *RTS1*-RFB being active or not, respectively. The percentage of deletion and translocation events, as determined by the PCR assay, was used to balance the rate of *ura4* loss. The values reported are means of at least three independent median rates ± standard deviation (SD). Statistically significant fold differences in the rates of deletion or translocation events from the *wt* strain are indicated with a asterisk (*p*<0.01). Statistical significance was calculated using the nonparametric Mann–Whitney U test. Refer to Data S1, sheet 1.

We then made use of another construct, *t> ura4 <ori*, that contains two *RTS1* sequences integrated at both sides of *ura4* (Figure 2A) [48]. A third *RTS1* sequence is present at its natural location, near the *mat* locus on the chromosome II. Given the orientation of the *RTS1* sequences relative to the main replication direction of each locus, the *RTS1* sequence at the centromere (*cen*)-proximal side of *ura4* behaves as a strong RFB, whereas the two other *RTS1* sequences have poor RFB activity. Occasionally during replication restart (in ∼2%–3% of the cell population/generation), HR-mediated template switch results in nascent strands inappropriately invading the *RTS1* sequence located in the vicinity of the blocked fork on chromosome III or the one located further away on chromosome II. Such faulty template switches lead to chromosomal rearrangements including inversions and large palindromic chromosomes, as well as the loss of the *ura4* marker (Figures 2A and S2A) [3],[48],[53]. Importantly, chromosomal rearrangements were also observed in response to MMS treatment and in the absence of active *RTS1*-RFB, showing that conditional fork barriers are relevant models for the generation of rearrangements initiated by template switch [7]. Also, replication restart and template switch-mediated rearrangements occur independently of PRR, making the *RTS1*-RFB assay particularly useful to decipher the role of CAF-1 in replication-coupled HR [7],[53].

### CAF-1 Promotes HR-Mediated Faulty Template Switch Resulting in Genome Rearrangements

In the *t> ura4 <ori* strain, fork arrest at the *cen*-proximal side of *ura4* leads to an 11-fold increase in the loss of the *ura4* marker (Table 1 and [53]). The loss of *ura4* corresponds to the deletion of *ura4* on chromosome III (genomic deletion) or translocation to chromosome II; each event can be distinguished by PCR (Figure 2A–B). Genomic deletion and translocation result from HR-mediated faulty template switches between the three dispersed *RTS1* sequences (Figure 2A) [53]. Consistent with this, both fork-arrest–induced genomic deletion and translocation are dependent on Rad52 (Table 1, Figure 2B, and [53]). The *rad52-d* mutant experiences a loss of viability upon induction of the *RTS1*-RFB, a phenotype not observed in strains deficient for individual CAF-1 subunits (Figure 2C). However, we observed that a defect in CAF-1 leads to a 3- to 5-fold reduction in the rate of fork-arrest–induced *ura4* loss, compared to the *wt* strain (Table 1). PCR analysis showed that both genomic deletion and translocation induced by the active *RTS1*-RFB were affected: In the *pcf1-d* mutant, these events were reduced by 6- and 10-fold, respectively, compared to the *wt* strain (*p*<0.0001) (Figure 2B–D). Our data indicate that, surprisingly, faulty template switch at blocked forks requires CAF-1. As Rad52 is efficiently recruited to the *RTS1*-RFB in the absence of CAF-1 (Figure S1D), this suggests that CAF-1 promotes template switch downstream of the recruitment of HR factors.

**Table 1:** Rate of *ura4* loss.

Strains	Rate of *ura4* Loss ×10^−8^ (Event/Cell/Generation)^a^	Fold Increase by RFB (OFF/ON)	Fold VARIATION over *wt* b
*wt* RFB OFF	1.6±0.2		
*wt* RFB ON	17.8±3.1	11.1	
*rad52-d* RFB OFF	120		Decreased by 8.5 (*p*<0.01)
*rad52-d* RFB ON	161	1.3	
*pcf1-d* RFB OFF	2.1±0.8		Decreased by 5 (*p*<0.0001)
*pcf1-d* RFB ON	5.9±1.5	2.8	
*pcf2-d* RFB OFF	2.4±0.6		Decreased by 3.3 (*p*<0.003)
*pcf2-d* RFB ON	8.1±1.1	3.4	
*pcf3-d* RFB OFF	2.2±0.8		Decreased by 3 (*p*<0.01)
*pcf3-d* RFB ON	8.2±0.3	3.7	
*pcf1-PIP^mut^* RFB OFF	2.3±0.3		Decreased by 3.8 (*p*<0.00003)
*pcf1-PIP^mut^* RFB ON	6.7±0.9	2.9	
*rqh1-d* RFB OFF	7.3±1.4		Increased by 2.9 (*p*<2.10^-6^)
*rqh1-d* RFB ON	232±6.5	31.8	
*rqh1-d pcf1-d* RFB OFF	6.3±1.4		Increased by 1.3 (*p*<0.01)
*rqh1-d pcf1-d* RFB ON	92.1±8.7	14.6	

a The values reported are means of at least three independent median rates ±SD.

b Statistical significance was calculated using the nonparametric Mann–Whitney test.

### CAF-1 Promotes Template Switch at Replication Forks by Stabilizing Rad51-Dependent D-Loops

**Figure 3:**
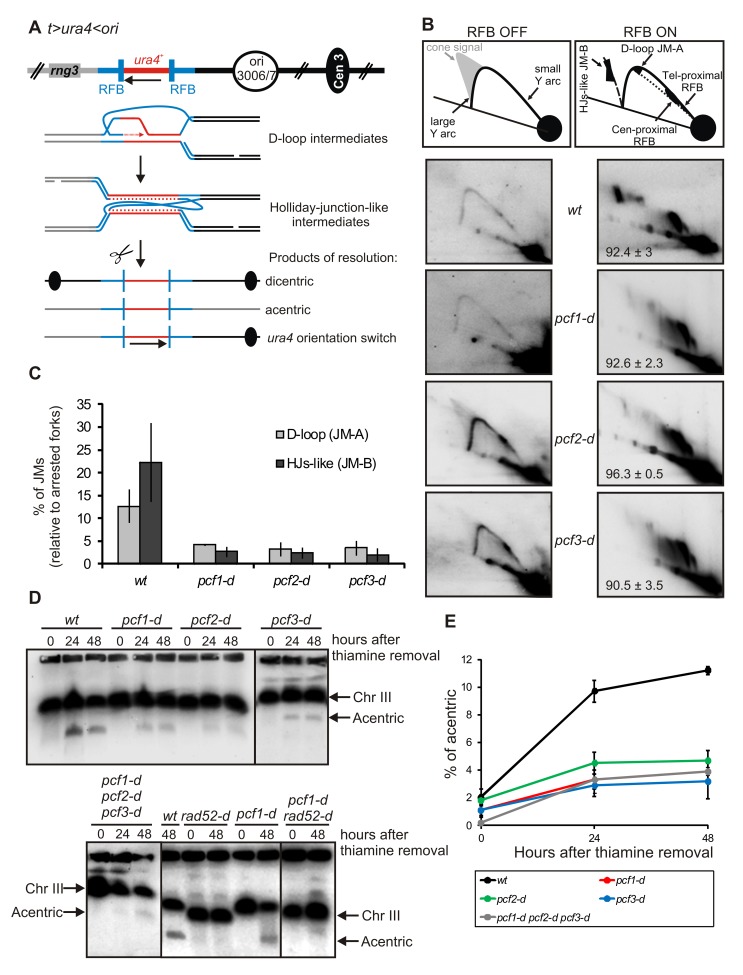
CAF-1 mediates template switch by stabilizing D-loop intermediates. (A) Diagram of the *t> ura4 <ori* locus (see Figure 2 legend for details). Upon fork arrest at the *RTS1*-RFB, stalled nascent strands switch template and invade the opposite *RTS1* sequence, leading to the formation of an early JM (D-loop, JM-A). The incoming of the opposite fork leads to a reciprocal template switch of stalled nascent strands leading to the formation of a late JM containing HJs (JM-B). The resolution of HJ-like structures leads to three distinct products: acentric or dicentric isochromosomes and the inversion of *ura4* orientation (indicated by a black arrow). (B) Analysis of RIs by 2DGE in indicated strains and conditions; ON and OFF refers to the *RTS1*-RFB being active or not, respectively. Top panels are diagrams of RIs within the A*se*1 restriction fragment analyzed by 2DGE in indicated conditions. Numbers ±SD, percentage of forks arrested at the *RTS1*-RFB. (C) Quantification of panel B. Values are the mean of three independent experiments ± standard error of the mean (SEM). Refer to Data S1, sheet 2. (D) Chromosomes from indicated strains and conditions were separated by PFGE and analyzed by Southern blotting using *rng3* probe, located *tel* proximal from *ura4*. Cells were grown with (RFB OFF, time 0) or without thiamine (RFB ON) for 24 and 48 h. (E) Quantification of the amounts of acentric chromosomes seen in panel D. Values correspond to the mean of at least three independent experiments ±SEM. Refer to Data S1, sheet 3.

In the *t> ura4 <ori* strain, fork arrest at the *cen*-proximal side of *ura4* induces stalled nascent strands to switch template and to recombine with the opposite *RTS1* sequence located on the *telomere*-proximal side of *ura4* (Figure 3A) [3]. This template switch event leads to a stable and early JM (JM-A), which consists of a D-loop structure. Then, the approaching opposite fork is stalled by the *RTS1*-RFB, leading to a second template exchange and the formation of a later JM (JM-B), which contains HJ-like structures (Figure 3A–B). Thus, the D-loop structure is the precursor of the HJ-like intermediate. Both types of JMs are detectable by 2DGE and are dependent on Rad52 [3]. The resolution of HJ-like structures leads to chromosomal rearrangements: acentric and dicentric isochromosomes or chromosomes in which *ura4* has switched orientation (Figures 3A and S2A). Chromosomal rearrangements can be detected by pulse field gel electrophoresis (PFGE) and restriction fragment length analysis (RFLA), followed by Southern blotting [3].

We further investigated the role of CAF-1 during template switch by these physical assays. The intensity of both types of JMs was severely decreased in strains deficient for individual CAF-1 subunits (Figure 3B–C). In addition, all types of chromosomal rearrangements, the products of resolution of HJ-like structures, were reduced by 2- to 3-fold in CAF-1 defective strains (*p*<0.003) (Figures 3D–E and S2B–C). Thus, the decreased intensity of HJ-like structures could not be explained by a faster cleavage of these structures. Because CAF-1 does not prevent HR factor recruitment at blocked forks, we rather envisioned that D-loop intermediates are formed but dismantled more quickly in the absence of CAF-1, thus resulting in a decreased level of HJ-like intermediates. To test this hypothesis, we analyzed genetic interactions with *mus81* (SPCC4G3.05c). Fission yeast Mus81 is an endonuclease involved in the cleavage of HJs [54]. As previously reported, HJ-like structures, but not D-loop intermediates, accumulated in *mus81-d* cells, thus resulting in cell death upon induction of the *RTS1*-RFB (Figure S3) [3]. Consistent with a faster dismantling of the D-loop and less HJ-like structures being produced in the absence of CAF-1, the deletion of *pcf1* rescued the sensitivity of *mus81-d* cells to the induction of the *RTS1*-RFB (Figure S3A–B). Analysis of JMs by 2DGE confirmed that the intensity of both JMs remained decreased in the double mutant compared to the single mutant *mus81-d* (Figure S3C–D). The data are consistent with the hypothesis that HJ-like structures are formed less often in the absence of CAF-1 due to faster dismantling of D-loop intermediates.

**Figure 4:**
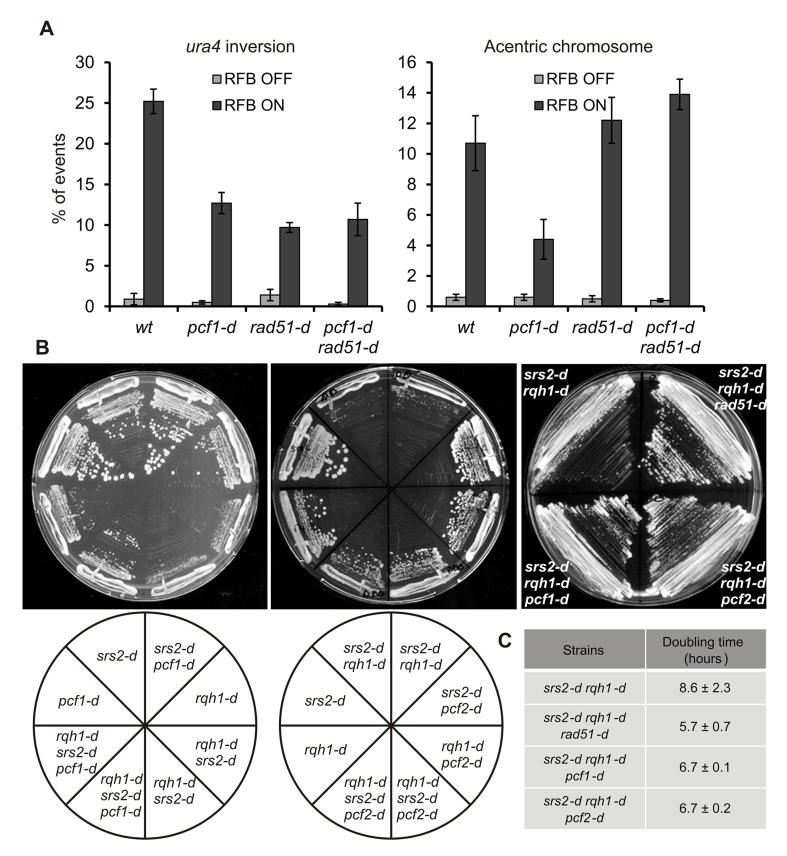
CAF-1 stabilizes Rad51-dependent JMs. (A) Quantification of *ura4* inversion and acentric chromosome in indicated strains and conditions (see Figure S2 for details). Values correspond to the mean of at least three independent experiments ±SD. Refer to Data S1, sheet 4. (B) Cell growth of indicated strains on rich media. (C) Table of doubling time from indicated strains in hours. Values are the mean of three independent experiments ±SD.

To reinforce this last hypothesis, we investigated genetic interaction with the recombinase *rad51* required to promote D-loop formation. In the absence of Rad51, chromosome rearrangements are produced without D-loop formation, probably via the single strand annealing function of Rad52 [3]. We reasoned that if CAF-1 stabilizes Rad51-dependent D-loop intermediates, its function in promoting template switch should rely on a functional Rad51 pathway. The type and level of chromosome rearrangements observed in the double mutant *pcf1-d rad51-d* was similar to those of the single *rad51-d* mutant, showing that *rad51* and *pcf1* are epistatic (Figures 4A and S4A–B). The data are consistent with CAF-1 acting in the Rad51 pathway to promote HR at replication forks, likely downstream of the formation of D-loop intermediates.

To extend our conclusion of CAF-1 acting in the Rad51 pathway to prevent D-loop disassembly, we performed genetic analysis. In both fission and budding yeast models, the concomitant inactivation of a RecQ-helicase and Srs2 results in a pronounced slow growth phenotype or cell death, a phenotype rescued by the deletion of *rad51* [47],[55],[56]. It has been proposed that this synthetic sickness/lethality results from the accumulation of unresolved JMs that impinge on cell fitness. Deleting either *pcf1* or *pcf2* led to a marked rescue of the slow growth phenotype of the *rqh1-d srs2-d* strain, although to a less extent than the deletion of *rad51* (Figure 4B–C). These data are consistent with CAF-1 acting in the Rad51 pathway and promoting template switch at replication forks by stabilizing D-loop intermediates.

### CAF-1 Counteracts D-Loop Disassembly by the RecQ-Type Helicase Rqh1

We investigated the mechanism by which CAF-1 prevents D-loop dismantling. Several helicases have been implicated in D-loop dissociation including Srs2, Fml1, and Rqh1 [15]–[23]. We found no evidence of Fml1 (SPAC9.05) promoting template switch at the site-specific arrested fork (unpublished data). Given the synergistic sensitivity of the double mutant *pcf1-d srs2-d* to MMS, we first analyzed the interactions between CAF-1 and Srs2 using our model system for template switch. We previously reported that Srs2 promotes JM formation and chromosomal rearrangements formed by template switch [3]. We found that strains defective for both CAF-1 and Srs2 showed a reduced level of chromosome rearrangements, similar to those observed in each single mutant (Figure S4). Thus, CAF-1 and Srs2 might act in the same pathway promoting template switch at replication forks.

**Figure 5:**
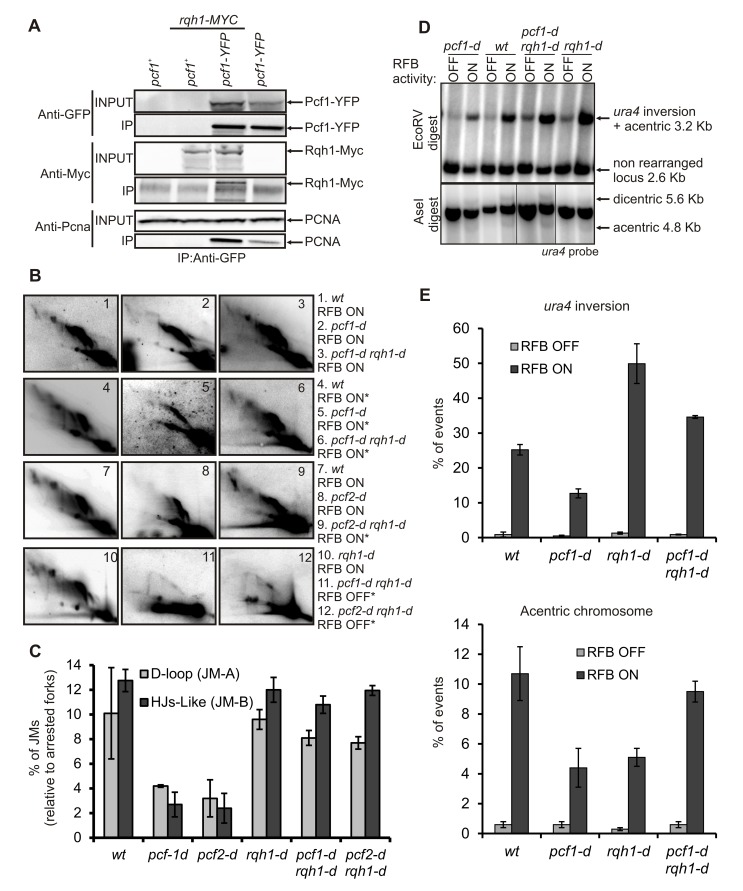
CAF-1 counteracts D-loop dismantling by the RecQ helicase Rqh1. (A) CAF-1 and Rqh1 physically interact. Immunoprecipitation of Pcf1-YFP by anti-GFP antibody in indicated strains. After protein separation by electrophoresis, samples were analyzed using anti-GFP antibody to reveal Pcf1-YFP, using anti-Myc to reveal Rqh1-MYC, and anti-PCNA. (B) Analysis of RIs by 2DGE in indicated strains and conditions; ON and OFF refers to the *RTS1*-RFB being active or not, respectively. Stars indicate DNA samples that have been cross-linked prior to extraction. Panels 1 to 3, 8, and 10 were done in the same set of experiments and panels 4 to 6 and 9 in another set of experiments. (C) Quantification of panel B. Values are the means of three independent experiments ±SD. Refer to Data S1, sheet 5. (D) Analysis of chromosomal rearrangements by Southern blotting using *ura4* probe (see Figure S2 for details). Indicated strains were grown with (RFB OFF) or without thiamine (RFB ON) for 48 h. Restriction enzymes and the origin of each signal are indicated. (E) Quantification of panel D in indicated strains. Values are the means of at least three independent experiments ±SD. Refer to Data S1, sheet 6.

The human RecQ helicase BLM and the large subunit of CAF-1 (p150) physically interact to coordinately promote cell survival to replication stress [57]. We found that the double mutant *pcf1-d rqh1-d* was more sensitive to MMS than the single *rqh1-d* mutant, *rqh1* (SPAC2G11.12) being the fission yeast homologue of *BLM* (Figure 1). Also, *pcf1-d rqh1-d* was more sensitive to camptothecin (CPT, a topoisomerase 1 inhibitor) than each single mutant, whereas the deletion of *pcf1* suppressed the sensitivity of the single mutant *rqh1-d* to hydroxyurea (HU), a ribonucleotide reductase inhibitor that depletes dNTP pools and stalls replication forks (Figure S5). Co-immunoprecipitation experiments showed that Rqh1 and Pcf1 physically interact (Figures 5A and S6A). Thus, functional interactions between CAF-1 and Rqh1 to promote cell resistance to replication stress are evolutionarily conserved. RecQ helicases prevent genome instability by promoting the dissolution of early (D-loop) and late (double HJs) JMs [54]. We previously have proposed that Rqh1 limits genome instability at replication forks by disassembling Rad51-dependent D-loops [3]. In the *RTS1*-RFB assay, HJs formed between *RTS1* repeats cannot branch migrate *in vitro* and thus cannot be resolved by dissolution. Accordingly, HJ-like intermediates did not accumulate in *rqh1-d* cells compared to *wt* cells (Figure 5B–C, panels 7 and 10) [3]. We analyzed whether Rqh1 could be responsible for D-loop dismantling in the absence of CAF-1. In a *pcf1-d rqh1-d* and in a *pcf2-d rqh1-d* strain, the level of both JMs was restored to those observed in either *rqh1-d* or *wt* cells (Figure 5B–C). To verify that the stability of JMs are restored *in vivo* and does not result from an *in vitro* artifact during DNA manipulation, DNA samples were cross-linked prior to extraction. In such conditions, the lack of JMs in the absence of CAF-1 was confirmed (Figure 5B, panel 5), showing that JMs are unstable *in vivo*. Also, the intensity of JMs was restored to a *wt* level by deleting *rqh1* (Figure 5B, panels 6 and 9), showing that Rqh1 is responsible for the lack of JMs in the absence of CAF-1 *in vivo*. Consistently, both *ura4* inversion and acentric chromosomes, the resolution products of HJ-like structures, were restored to *wt* levels in strains defective for CAF-1 and Rqh1 (Figures 5D–E and S6B). Our data establish that Rqh1 disassembles the D-loop in the absence of CAF-1, and we propose that CAF-1 promotes template switch at the replication fork by counteracting D-loop disassembly by Rqh1.

We analyzed the level of genomic deletion and translocation that result from faulty template switch between *RTS1* sequences on chromosomes II and III [53]. Following induction of the *RTS1*-RFB, deleting *pcf1* resulted in a 6 and 10 times reduction in the rate of genomic deletion and translocation, respectively, compared to the *wt* strain (Figure S6C–D). In contrast, the rate of these events was reduced by only ∼1.8 times by deleting *pcf1* in the absence of Rqh1 (Table 1 and compare *rqh1-d* and *pcf1-d rqh1-d* strains on Figure S6C). PCR analysis showed that translocation and deletion events were decreased in *pcf1-d* cells compared to *wt* cells (Figure 2B), but not when *rqh1* is deleted (Figure S6D). Altogether our data reveal that the likelihood of faulty template switch is controlled by the antagonistic roles of CAF-1 and Rqh1 in processing the D-loop.

### Stabilization of the D-Loop by CAF-1 Requires the Full Complex and Its Interaction with PCNA

CAF-1 mediates replication-coupled chromatin assembly and interacts with the heterochromatin factor Swi6 (SPAC664.01c, the human HP1 homologue) to assist the maintenance of heterochromatin and silencing during S-phase [29]. A strain mutated for *swi6* exhibited no defect in the accumulation of the acentric chromosome following the activation of the *RTS1*-RFB, indicating that the role of CAF-1 in template switch is unlikely to involve heterochromatin (Figure S7).

Deposition of histone H3-H4 onto newly synthesized DNA by CAF-1 requires, *in vitro*, its three subunits and its ability to interact with the replication factor PCNA (SPBC16D10.09) [28],[31]–[33],[35],[37],[44]. The three strains *pcf1-d*, *pcf2-d*, and *pcf3-d* exhibited a similar phenotype: fewer faulty template switches and a faster dismantling of the D-loop (Figures 2 and 3). A strain in which the three subunits of CAF-1 have been inactivated showed a decreased level of acentric chromosomes, one of the products of JM resolution, similar to those observed in each single mutant (Figure 3D–E). Thus, the role of CAF-1 in promoting template switch is not specific to a single subunit but necessitates the three subunits to act in the same HR pathway.

**Figure 6:**
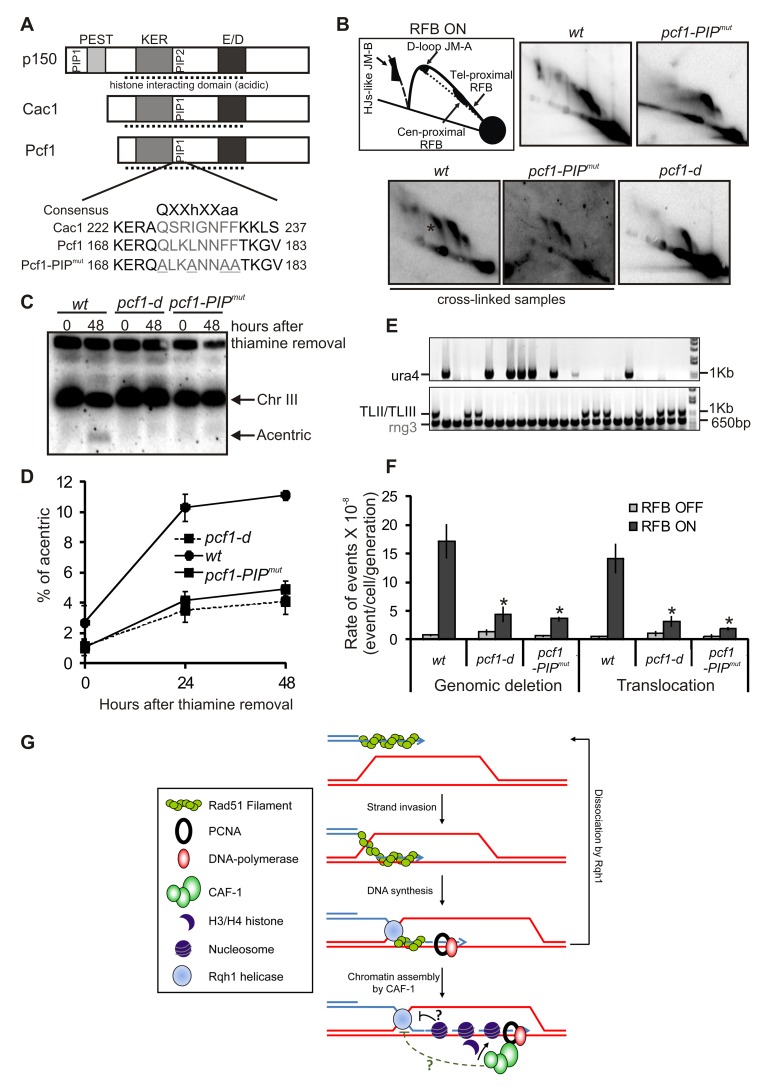
Preventing D-loop disassembly by CAF-1 requires its ability to interact with PCNA. (A, Top panel) Alignment of the human (p150), *S. cerevisiae* (Cac1), and *S. pombe* (Pcf1) large subunit of CAF-1. Boxes indicate the PEST, KER, and E/D domains. The dashed line indicates the acidic region involved in histone binding. PIP1 and 2 indicate the PCNA interacting peptide. (Bottom panel) PIP1 sequence in Cac1 and Pcf1. Numbers refer to amino acids. Underlined amino acids indicate mutation introduced in the Pcf1-PIP^mut^ protein. (B) Analysis of RIs by 2DGE in indicated strains and conditions; ON and OFF refers to the *RTS1*-RFB being active or not, respectively. Top panels are diagrams of RIs within the A*se*1 restriction fragment analyzed by 2DGE in indicated conditions. Stars indicate additional signals resulting from partial digestion due to cross-linked DNA. (C) Chromosomes from indicated strains and conditions were separated by PFGE and analyzed by Southern blotting using *rng3* probe, located *tel* proximal from *ura4*. Cells were grown with (RFB OFF) or without thiamine (RFB ON) for 48 h. (D) Quantification of the amounts of acentric chromosomes seen in panel C. Values correspond to the mean of at least three independent experiments ±SEM. Refer to Data S1, sheet 7. (E) Representative PCR amplification from 5-FOA^R^ colonies from the indicated strain and condition. PCR products and their sizes are indicated on the figure. (F) Rate of genomic deletion and translocation for the strains indicated; ON and OFF refers to the *RTS1*-RFB being active or not, respectively. The percentage of deletion and translocation events, as determined by the PCR assay, was used to balance the rate of *ura4* loss. The values reported are means of at least three independent median rates ±SD. Statistically significant fold differences in the rates of deletion or translocation events from the *wt* strain are indicated with an asterisk (*p*<0.01). Statistical significance was calculated using the nonparametric Mann–Whitney U test. Refer to Data S1, sheet 8. (G) Model of D-loop stabilization by CAF-1 during template switch. CAF-1 might prevent the disassembly of the D-loop by promoting histone deposition coupled to DNA synthesis. Nascent chromatin assembled on the D-loop then counteracts Rqh1 activity (black line). Alternatively, CAF-1 is targeting on the D-loop via its interaction with PCNA and counteracts the activity of Rqh1 directly or indirectly (dashed green line).

In budding and fission yeast, the large subunit of CAF-1 contains only one canonical PCNA interacting peptide (PIP box). We mutated the key residues to alanine to generate a mutant of *pcf1* unable to interact with PCNA (*pcf1-PIP^mut^*) (Figure 6A). Co-immunoprecipitation showed that mutating the PIP box of Pcf1 severely impaired the interaction of Pcf1 with PCNA without affecting its interaction with Pcf2 (Figure S8A). The interaction of Pcf2 with PCNA was also dependent on the PIP box of Pcf1 (Figure S8A). Thus, expressing Pcf1-PIP^mut^ leads to the formation of a CAF-1 complex unable to interact with PCNA. As expected, mutating the PIP box of Pcf1 led to a loss of Pcf1 foci in S-phase cells and a loss of co-localization with replication factories (labeled with a CFP-tagged version of PCNA) (Figure S8B). Thus, the canonical PIP box of Pcf1 is sufficient to target CAF-1 into replication foci and expressing Pcf1-PIP^mut^ is likely to impair replication-coupled chromatin assembly by CAF-1. Then, we investigated the phenotype of the *pcf1-PIP^mut^* strain. First, the stability of JMs was impaired and consistently the level of the acentric chromosome (one of the products of JM resolution) was reduced as in the *pcf1-d* strain (Figure 6B,C,D). Second, the rates of genomic deletion and translocation induced by the active *RTS1*-RFB were similarly decreased in *pcf1-PIP^mut^* and *pcf1-d* cells, compared to the *wt* strain (Table 1, Figure 6E–F). Thus, mutating the PIP box of Pcf1 is sufficient to mimic the deletion of Pcf1. Thus, the role of CAF-1 in promoting template switch by preventing Rqh1-dependent dismantling of the D-loop requires the full complex and the capacity to interact with PCNA.

## Discussion

We have discovered a novel chromatin-factor–dependent step during HR-mediated template switch, involving CAF-1: the protection of the D-loop from disassembly by the RecQ-type helicase Rqh1. First, CAF-1 promotes an HR-dependent and replication-coupled repair pathway, independently of the error-prone and error-free branch of PRR. Second, using a genetic assay that selects for recombination events at replication forks, we establish that CAF-1 promotes template switch by counteracting D-loop disassembly by Rqh1. Third, Rqh1 and CAF-1 physically interact. Consequently, the likelihood of faulty template switch is controlled by the opposite activities of CAF-1 and Rqh1 in processing the D-loop. Finally, the D-loop protection by CAF-1 requires the full complex and its interaction with PCNA, but not the heterochromatin factor Swi6. Our data are thus consistent with a model in which D-loop extension by DNA synthesis is coupled to histone deposition by CAF-1. We propose that the newly assembled nucleosomes on the D-loop display a substrate less favorable to antirecombinase Rqh1 action, thus protecting the D-loop from being dismantled (Figure 6G, black line). This pathway does not exclude other mechanisms such as a negative interference of CAF-1 with Rqh1 activity either directly or via an additional nonhistone factor. This second mechanism would also require PCNA-mediated localization of the CAF-1 complex during D-loop extension (Figure 6G, dashed green line).

### CAF-1 Acts in the Rad51-Dependent Pathway to Promote Replication-Coupled DNA Repair by Stabilizing D-Loop Intermediates

Beyond its role in chromatin restoration at DNA damage sites, roles for CAF-1 in recombinational DNA repair pathways have been reported [24],[45],[46]. Budding yeast CAF-1 protects against DSBs by acting both in HR and nonhomologous end-joining pathways [58],[59]. A defect in CAF-1 also leads to a decreased efficiency of DSB-induced recombinational repair in drosophila [60]. More recently, a genetic screen has identified CAF-1 as promoting break-induced replication, a one-ended invasion HR pathway that occurs when the homology between the broken end and the donor DNA molecules is limited to one broken arm [61]. In mammals, CAF-1 acts in both the early and late steps of HR-mediated DNA repair by promoting the resection of DSBs and the recruitment of HR factors and then the restoration of chromatin state when repair is completed [24].

Here, we report that CAF-1 promotes replication-coupled DNA repair independently of the error-prone and error-free branches of PRR. The sealing of ssDNA gaps left behind moving forks involves template switches mediated by the error-free branch of PRR [4],[5]. This damage tolerance pathway requires Rad5, Rad51, and the ubiquitination of PCNA. Our data place CAF-1 in an alternative Rad51-dependent template switch pathway. Consistent with this, replication restart and chromosome rearrangements mediated by template switch at site-specific arrested forks occur independently of the ubiquitination of PCNA [7],[53]. We propose that CAF-1 acts in Rad51-dependent template switches occurring during replication restart. We identified the underlying mechanism: CAF-1 stabilizes the D-loop by preventing its disassembly by the helicase Rqh1. Consequently, the likelihood of faulty template switch, a type of NAHR causing chromosomal rearrangements, is controlled by the antagonistic activities of CAF-1 and Rqh1 at the D-loop: CAF-1 stabilizing the D-loop and Rqh1 promoting its disassembly. Functional interplays between CAF-1 and Rqh1 in response to replication stress are evolutionarily conserved (see below).

In mammals, CAF-1 primes HR events at DNA damage by promoting the end-resection of DSBs and thus the recruitment of HR factors such as Rad51 [24]. Then, CAF-1 might switch towards its histone chaperone mode to restore chromatin after the completion of the HR event. Here, we report a novel step at which CAF-1 promotes HR: By preventing D-loop disassembly, CAF-1 impacts the resolution of the subsequent HR event. Thus, the role of CAF-1 during HR might be more dynamic than previously anticipated, not only acting in the early and final steps, but having potential roles all along the HR process. We propose that CAF-1, and potentially chromatin assembly coupled to the DNA synthesis step of the HR event, is an important regulatory point of template switch.

### CAF-1 Likely Prevents Rqh1-Dependent D-Loop Disassembly Via Its Chromatin Assembly Function

Defects in the RecQ-type helicase BLM lead to Bloom's syndrome, a human disorder associating genomic instability and cancer predisposition. Functional interactions between BLM and the p150 large subunit of CAF-1 have been previously reported in response to replication stress [57]. Here, we identified that interplays between CAF-1 and BLM are evolutionarily conserved in fission yeast. The large subunit of CAF-1, Pcf1, and Rqh1 physically interact and act in a coordinated way to promote survival and maintain genome stability in response to replication stress. Importantly, we uncovered the underlying mechanism. Using genetic and physical assays that allow the analysis of the individual steps of HR-mediated template switch at a single replication fork, we found that the impaired stability of D-loop intermediates due to a CAF-1 defect results from the activity of Rqh1. CAF-1 thus counteracts D-loop dismantling by Rqh1. The RecQ helicase family is also involved in the rescue and stability of stalled forks, however we excluded interplays between CAF-1 and Rqh1 in this process [62]–[64]. First, the site-specific arrested fork is stable and prone to recombination events in both single and double mutants. Second, CAF-1 acts downstream of D-loop formation by Rad51.

We hypothesize that nucleosome assembly on the D-loop is promoted by the interaction of CAF-1 with PCNA and that the nucleosomal nature of the D-loop prevents disassembly by Rqh1. We cannot exclude that the interaction with PCNA simply serves to recruit CAF-1 to the D-loop where CAF-1 could either directly counteract Rqh1 action or trigger the recruitment of an additional factor counteracting Rqh1 activity (Figure 6G). In human cells, BLM inhibits CAF-1–mediated chromatin assembly coupled to DNA repair [57]. Through physical interactions, Rqh1 could also mediate CAF-1 recruitment to the D-loop on which Rqh1 could inhibit chromatin assembly by CAF-1. However, such hypotheses are not sufficient to account for all our observations. Indeed, in the absence of CAF-1 and of any potential histone deposition on JMs, the D-loop is disassembled faster by Rqh1, thus rather suggesting a model in which CAF-1 counteracts Rqh1 activity.

Preventing Rqh1-dependent D-loop dismantling requires the three subunits of CAF-1 and its interaction with PCNA as for optimal histone deposition onto newly replicated DNA *in vitro* [25],[31],[37]. Therefore, we propose that CAF-1 prevents D-loop disassembly by promoting histone deposition onto the D-loop. We could not confirm this hypothesis by generating CAF-1 mutated forms unable to interact with histones, as CAF-1 binds histone H3-H4 by multiple interactions: Each subunit interacts directly with histones and independently of the two other subunits. The human p150 interacts with histone H3-H4 via an acidic domain of 350 residues containing the KER and ED domains. The third subunit p48 interacts with the N-terminal domain of histone H4, and deleting this domain is not sufficient to abolish *in vitro* chromatin assembly [65]. Thus, the complexity of the protein interface between CAF-1 and histones currently limits our ability to genetically impair the interaction of CAF-1 with histones.

### CAF-1 Modulates Template Switch and Is Critical When the Homology Between DNA Molecules Is Limited

Although the sole absence of CAF-1 does not confer cell sensitivity to MMS, our data place CAF-1 in a Rad51-dependent template switch pathway by stabilizing D-loop intermediates. Thus, redundant pathways must exist for D-loop stabilization. On the other hand, CAF-1 is critical for faulty template switch events occurring between repeated sequences, a type of NAHR. Protection of the D-loop by CAF-1 during extension by DNA synthesis might provide a mechanism that allows the stabilization of the heteroduplex. This CAF-1–dependent D-loop stabilization might be critical when the homology between DNA molecules is limited (e.g., in NAHR), but alternative mechanisms of stabilizing the heteroduplex likely exist in the case of allelic HR. For example, the ability of Rad51 to branch migrate a single HJ behind the initial point of strand invasion provides the opportunity to extend the heteroduplex without DNA synthesis [66]. Such a mechanism can operate when the two recombinant molecules share a substantial length of homology. The confined length of homology in case of faulty template switch (∼900 bp for the *RTS1* sequence compared to unconfined length of homology between sister chromatids) might restrict the effectiveness of this process. Thus, CAF-1 modulates Rad51-dependent template switch, but because of alternative pathways to stabilize the D-loop, a defect in CAF-1 does not completely eliminate template switch. Further investigations are necessary to explore other mechanisms of D-loop stabilization.

In conclusion, CAF-1 promotes Rad51-mediated template switch events at replication forks by counteracting Rqh1-dependent D-loop dismantling. We propose that when CAF-1 switches towards its histone chaperone mode to promote histone deposition, this pathway impacts the resolution of the subsequent template switch event and thus genome stability. In mammals, HR is one of two replication fork maintenance pathways that fuse inverted repeats to mediate chromosome rearrangements, especially in the absence of BLM [11]. Given that functional interactions between CAF-1 and BLM in response to replication stress are evolutionarily conserved, it is possible that the role of CAF-1 in preventing D-loop disassembly is conserved in mammals and might account for the genetic instability associated with Bloom's syndrome.

## Materials and Methods

### Standard Genetic

Strains used were constructed by standard genetic techniques and are listed in Table S1.

The rate of *ura4* loss (presented in Table 1), genomic deletion, and translocation was determined as previously reported, as well as PCR analysis of 5-FOA–resistant cells [53]. Statistical significance was detected using the nonparametric Mann–Whitney U test.

### Molecular Biology

RIs were analyzed by 2DGE as reported [3]. Zymolyase-treated cells were embedded in agarose plug, treated with proteinase K, and washed several times in TE. After restriction digestion by A*se*I, RIs were enriched on BND cellulose columns, precipitated, and separated by 2DGE, according to [67], using 0.35% and 0.9% agarose gel for the first and second dimension, respectively. Quantification of RIs was performed as reported, using a phosphor-imager (Typhoon-trio) to detect ^32^P-probed signal. Briefly, fork termination and JM signal were quantified as a percentage of stalled fork signal. DNA samples were cross-linked using tri-methyl psoralen (Trioxsalen, Sigma) as follows: 2.10^9^ cells were washed twice in water and resuspended in 20 ml of cold water and placed into an 8.5-cm-diameter glass petri dish on ice. Cells were mixed with 1 ml of Trioxsalen at 200 µ/ml, incubated on ice for 5 min in the dark, and mixed every minute. Cells were then exposed to UV-A (365 nm) for 90 s at a flow of 50 mW/cm^2^. Analysis of restriction fragments by electrophoresis in denaturing conditions showed that 2–3 inter-crosslinks were formed every 500 bp (unpublished data).

Chromosomal rearrangements were analyzed by PFGE or Southern blot as previously reported [3],[48]. Rad52-GFP enrichment at the *RTS1*-RFB was performed as previously reported using the primer listed in Table S2 and using a polyclonal anti-GFP antibody (A11122 from Life Technologies) [48].

For immunoprecipitation experiments, the following procedure was used, based on the method published by [29]: 5.10^8^ cells were washed in cold water, resuspended in 400 µl of EB buffer (50 mM HEPES High salt, 50 mM KOAc pH 7.5, 5 mM EGTA, 1% triton X-100, 1 mM PMSF, and anti-protease), ribolysed with glass beads, and centrifuged. The supernatant was recovered and an aliquot of 50 µl was kept as INPUT control. Then, 2 µl of anti-GFP (A11122 from Life Technologies) or anti-Myc (c-Myc 9E10: sc-40 from Santa Cruz Biotechnology) antibody was added to 300 µl of the supernatant and incubated for 1 h at 4°C on a wheel. Then, 40 µl of prewashed Dynabeads protein-G (Life Technologies) was added and then incubated at 4°C overnight. Beads were then washed twice 10 min in EB buffer before separating proteins on acrylamide gel for analysis by Western blot with appropriate antibodies.

### Cell Biology

Cells were grown in filtered minimal medium (EMM) containing glutamate and implemented in amino acids and bases. Around 5.10^6^ to 1.10^7^ cells from an exponential culture were centrifuged at low speed (1,500 rpm for 1 min) and then resuspended in 1 ml of fresh filtered media. A drop of 1 µl was dropped onto a microscopy agarose slide containing a layer of 1.4% agarose dissolved in filtered media. Cells were observed with a LEICA DMRXA microscope equipped of an oil immersion 100× objective, with a numerical aperture of 1.4 and coupled to a COOLSNAP HQ camera (Roper Scientific, USA). The filters used were a FITC filter to collect GFP signal, CFP for CFP signal, and YFP for YFP signal. Images were taken with the Z-stack (3D) parameterized at 15 slices and were analyzed using METAMORPH (Roper Scientific, USA) and Image J software.

## Supporting Information

Figure S1 **Conditional fork barriers and HR factor recruitment at blocked forks are functional in the absence of CAF-1.** (A) Diagram of the *t-ura4 <ori* locus, in which *t* refers to the telomere (gray lines), <refers to the polarity of the *RTS1*-RFB (blue bars), and *ori* refers to the replication origin (opened black circle) on the centromere proximal side (the black circle indicates the centromere of chromosome III). In the absence of thiamine in the media, Rtf1 binds *RTS1* and mediates polar fork arrest at *ura4*. (B) 2DGE of RIs from indicated strains grown with the *RTS1*-RFB being induced (ON) or not (OFF). Top panels are diagrams of RIs within the A*se*1 restriction fragment analyzed by 2DGE in indicated conditions. Numbers ±SD, percentage of forks arrested at the *RTS1*-RFB. (C) Quantification of termination signal from panel B in indicated strains. Values are the means of three independent experiments ±SEM. Refer to Data S1, sheet 9. (D) qPCR analysis of Rad52 chromatin immune precipitation at the *RTS1*-RFB in indicated strains and conditions. Values are means of at least three independent experiments ±SEM. Refer to Data S1, sheet 10.
(TIF)

Figure S2 **Analysis of chromosomal rearrangements, the resolution products of HJ-like structures, in the absence of CAF-1.** (A) Diagram of the *t> ura4 <ori* locus and associated rearrangements, as indicated on Figure 3. A*se*1 and E*co*RV restriction fragment length are indicated in red and black, respectively. The left part indicates the size of restriction fragment for each rearrangement. (B) Analysis of chromosomal rearrangements by Southern blotting using *ura4* probe. Indicated strains were grown with (RFB OFF) or without thiamine (RFB ON) for 24 or 48 h. Restriction enzymes and the origin of each signal are indicated. (C) Quantification of panel B in indicated strains. Values are the mean of at least three independent experiments ±SEM. Refer to Data S1, sheet 11.
(TIF)

Figure S3 **D-loop intermediates are dissolved faster in the absence of CAF-1. (A) Survival of indicated strains upon fork arrest at the** ***t> ura4 <ori*** **locus.** Serial 10-fold dilution from indicated strains spotted onto media containing thiamine (RFB OFF) or not (RFB ON). (B) Quantification of panel A. Values are the mean of at least three independent experiments ±SD. Refer to Data S1, sheet 12. (C) Analysis of RIs by 2DGE in indicated strains upon activation of the *RTS1*-RFB. (D) Quantification of panel C. Values are the mean of three independent experiments ±SD. Refer to Data S1, sheet 13.
(TIF)

Figure S4 **Genetic interactions between CAF-1–defective strains,** ***rad51*** **and** ***srs2*** **.** (A) Chromosomes from indicated strains and conditions were separated by PFGE and analyzed by Southern blotting using *rng3* probe, located *tel* proximal from *ura4*. Cells were grown with (RFB OFF, time 0) or without thiamine (RFB ON) for 24 and 48 h. (B) Quantification of the amounts of acentric chromosomes seen in panel A. Values correspond to the mean of at least three independent experiments ±SEM. Refer to Data S1, sheet 14. (C) Quantification of *ura4* inversion and acentric chromosome in indicated strains and conditions (see Figure S2 for details). Values correspond to the mean of at least three independent experiments ±SD. Refer to Data S1, sheet 15.
(TIF)

Figure S5 **Genetic interactions between** ***rqh1*** **and** ***pcf1*** **in response to replication stress.** Serial 10-fold dilution from indicated strains spotted onto media containing indicated hydroxyurea (HU, top panel) or camptothecin (CPT, bottom panel) concentration.
(TIF)

Figure S6 **Functional and physical interactions between Pcf1, the large subunit of CAF-1, and Rqh1.** (A) Immunoprecipitation of Pcf1-YFP by anti-GFP antibody in indicated strains. After protein separation by electrophoresis, samples were analyzed using anti-GFP antibody to reveal Pcf1-YFP, using Rqh1 antibody (a gift from J. Murray) to reveal endogenous Rqh1, and anti-PCNA. (B, Top panel) Chromosomes from indicated strains and conditions were separated by PFGE and analyzed by Southern blotting using Rng3 probe, located *tel* proximal from *ura4*. Cells were grown with (RFB OFF) or without thiamine (RFB ON) for 24 or 48 h. (Bottom panel) Quantification of the amounts of acentric chromosomes. Values correspond to the mean of at least three independent experiments ±SEM. Refer to Data S1, sheet 16. (C) Rate of genomic deletion and translocation for the strains indicated; ON and OFF refers to the *RTS1*-RFB being active or not, respectively. The percentage of deletion and translocation events, as determined by the PCR assay, was used to balance the rate of *ura4* loss. The values reported are means of at least three independent median rates ±SD. Statistical significance was calculated using the nonparametric Mann–Whitney U test. Refer to Data S1, sheet 17. (D) Representative PCR amplification from 5-FOA^R^ colonies from indicated strains and conditions. PCR products and their sizes are indicated on the figure.
(TIF)

Figure S7 **The heterochromatin factor Swi6 is not involved in template switch.** (A) Chromosomes from indicated strains and conditions were separated by PFGE and analyzed by Southern blotting using Rng3 probe, located *tel* proximal from *ura4*. Cells were grown with (RFB OFF) or without thiamine (RFB ON) for 24 and 48 h. (B) Quantification of the amounts of acentric chromosomes seen in panel A. Values correspond to the mean of at least three independent experiments ±SEM. Refer to Data S1, sheet 18.
(TIF)

Figure S8 **CAF-1 and PCNA interaction targets CAF-1 in replication foci.** (A, Left panel) Immunoprecipitation of Pcf1-YFP by anti-GFP antibody in indicated strains. After protein separation by electrophoresis, samples were analyzed using anti-GFP antibody to reveal Pcf1-YFP, or Pcf1PIP^mut^-YFP, using anti-Myc to reveal Pcf2-MYC, and anti-PCNA. The mutation of the PIP box of Pcf1 severely impaired Pcf1/PCNA interaction without affecting Pcf1/Pcf2 interaction. (Right panel) Immunoprecipitation of Pcf2-MYC by anti-Myc antibody in indicated strains. After protein separation by electrophoresis, samples were analyzed using anti-Myc to reveal Pcf2-MYC, and anti-PCNA. The mutation of the PIP box of Pcf1 is sufficient to impair Pcf2/PCNA interaction. (B) Co-localization of PCNA-CFP and wt Pcf1-YFP (left panels) or Pcf1PIP^mut^-YFP (right panels). Differential interferential Contrast (DIC), CFP (Cyan), YFP (Yellow), and overlay acquisition signals are presented. The scale is indicated on the figure. Red arrows indicate S-phase cells (septated cells). White and yellow arrows indicate examples of S-phase nuclei containing PCNA or Pcf1 foci, respectively.
(TIF)

Table S1 **Strains used in this study.**
(DOCX)

Table S2 **Primers used for ChIP/qPCR.**
(DOCX)

Data S1 **Raw data used to derive the results contained in this study.**
(XLSX)

## Abbreviations

2DGE: bidimensional gel electrophoresis
CAF-1: chromatin assembly factor 1
CO: crossover
CPT: camptothecin
HJ: Holliday junction
HR: homologous recombination
HU: hydroxyurea
NAHR: nonallelic homologous recombination
PFGE: pulse field gel electrophoresis
RFB: replication fork barrier
