# Supplementary figures and images for: The Chromatin Assembly Factor 1 Promotes Rad51-Dependent Template Switches at Replication Forks by Counteracting D-Loop Disassembly by the RecQ-Type Helicase Rqh1
Source: PLoS Biol. 2014 Oct 14;12(10):e1001968. doi: 10.1371/journal.pbio.1001968 (PMC4196752; doi:10.1371/journal.pbio.1001968)

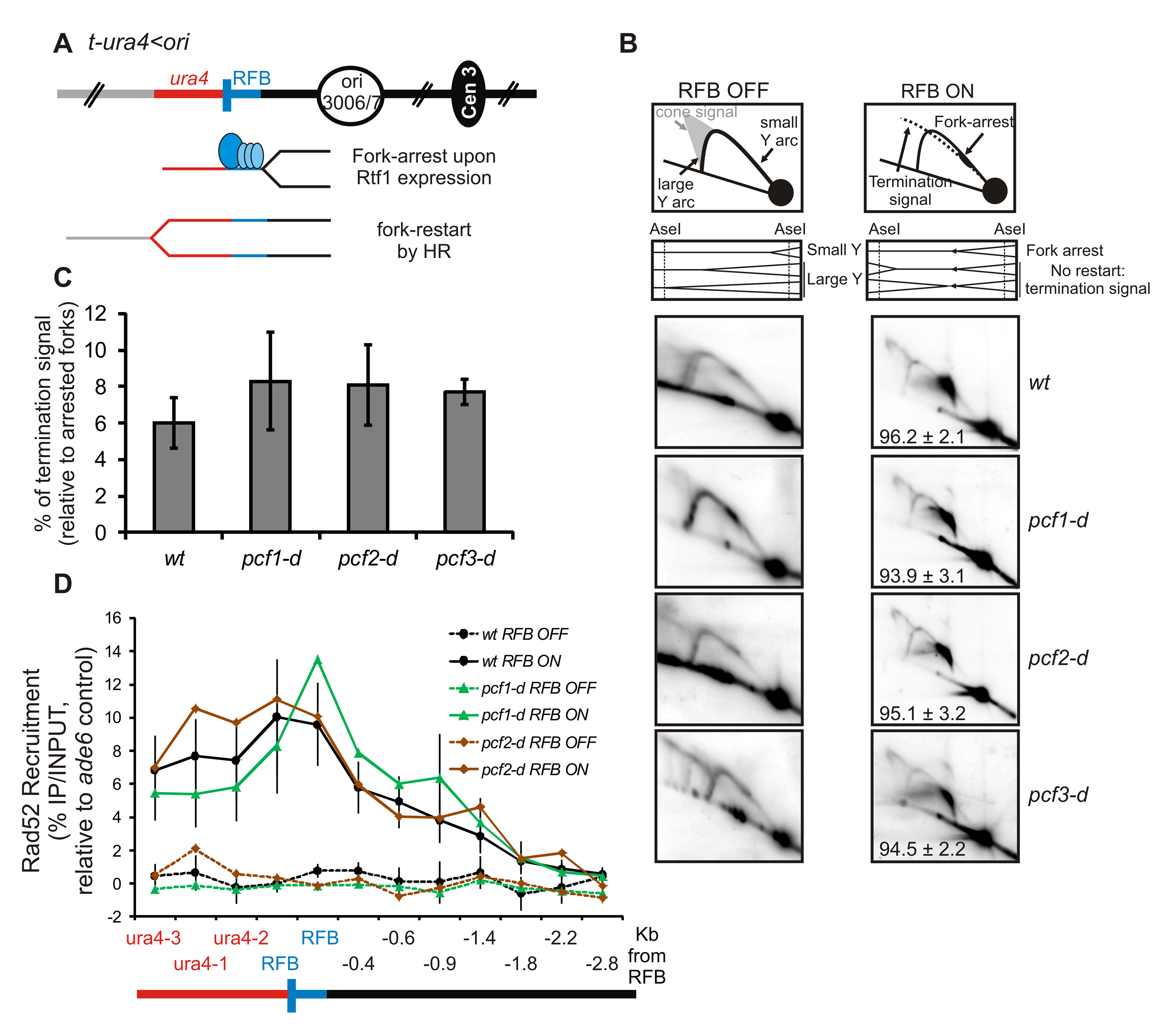

Supplement: Figure S1 — Conditional fork barriers and HR factor recruitment at blocked forks are functional in the absence of CAF-1. (A) Diagram of the t-ura4 <ori locus, in which t refers to the telomere (gray lines), <refers to the polarity of the RTS1-RFB (blue bars), and ori refers to the replication origin (opened black circle) on the centromere proximal side (the black circle indicates the centromere of chromosome III). In the absence of thiamine in the media, Rtf1 binds RTS1 and mediates polar fork arrest at ura4. (B) 2DGE of RIs from indicated strains grown with the RTS1-RFB being induced (ON) or not (OFF). Top panels are diagrams of RIs within the Ase1 restriction fragment analyzed by 2DGE in indicated conditions. Numbers ±SD, percentage of forks arrested at the RTS1-RFB. (C) Quantification of termination signal from panel B in indicated strains. Values are the means of three independent experiments ±SEM. Refer to Data S1, sheet 9. (D) qPCR analysis of Rad52 chromatin immune precipitation at the RTS1-RFB in indicated strains and conditions. Values are means of at least three independent experiments ±SEM. Refer to Data S1, sheet 10. (TIF) [file pbio.1001968.s001.tif]

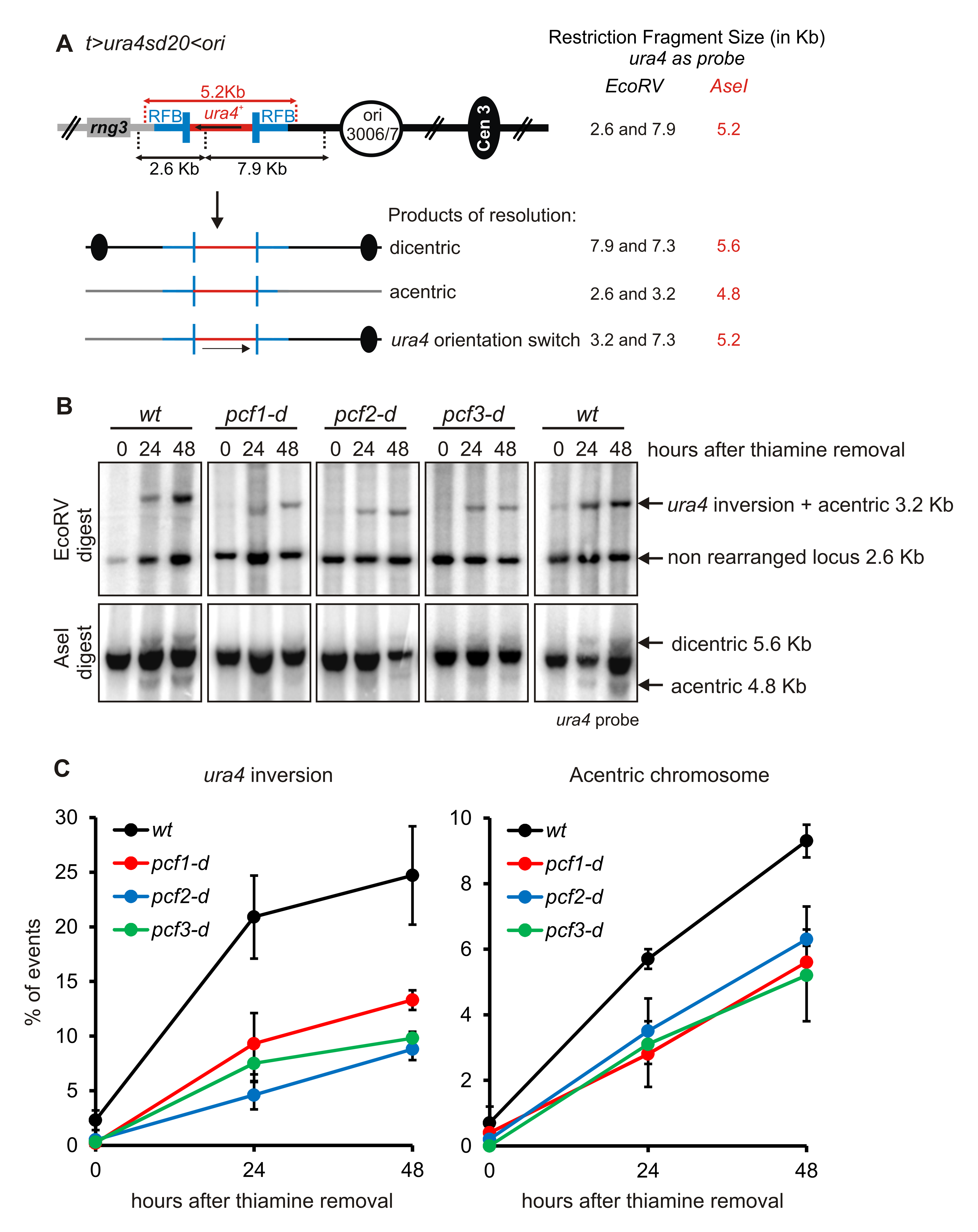

Supplement: Figure S2 — Analysis of chromosomal rearrangements, the resolution products of HJ-like structures, in the absence of CAF-1. (A) Diagram of the t> ura4 <ori locus and associated rearrangements, as indicated on Figure 3. Ase1 and EcoRV restriction fragment length are indicated in red and black, respectively. The left part indicates the size of restriction fragment for each rearrangement. (B) Analysis of chromosomal rearrangements by Southern blotting using ura4 probe. Indicated strains were grown with (RFB OFF) or without thiamine (RFB ON) for 24 or 48 h. Restriction enzymes and the origin of each signal are indicated. (C) Quantification of panel B in indicated strains. Values are the mean of at least three independent experiments ±SEM. Refer to Data S1, sheet 11. (TIF) [file pbio.1001968.s002.tif]

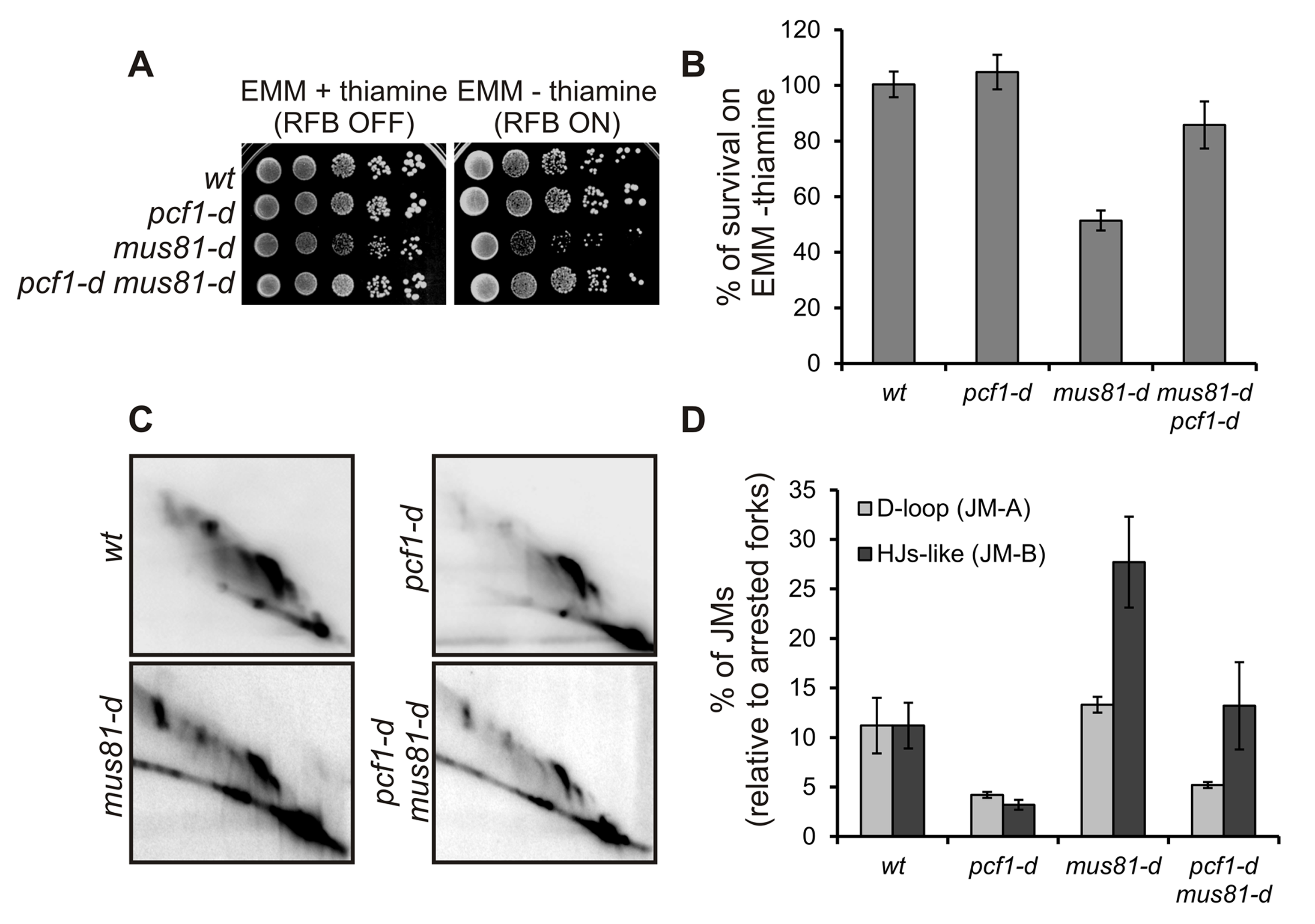

Supplement: Figure S3 — D-loop intermediates are dissolved faster in the absence of CAF-1. (A) Survival of indicated strains upon fork arrest at the t> ura4 <ori locus. Serial 10-fold dilution from indicated strains spotted onto media containing thiamine (RFB OFF) or not (RFB ON). (B) Quantification of panel A. Values are the mean of at least three independent experiments ±SD. Refer to Data S1, sheet 12. (C) Analysis of RIs by 2DGE in indicated strains upon activation of the RTS1-RFB. (D) Quantification of panel C. Values are the mean of three independent experiments ±SD. Refer to Data S1, sheet 13. (TIF) [file pbio.1001968.s003.tif]

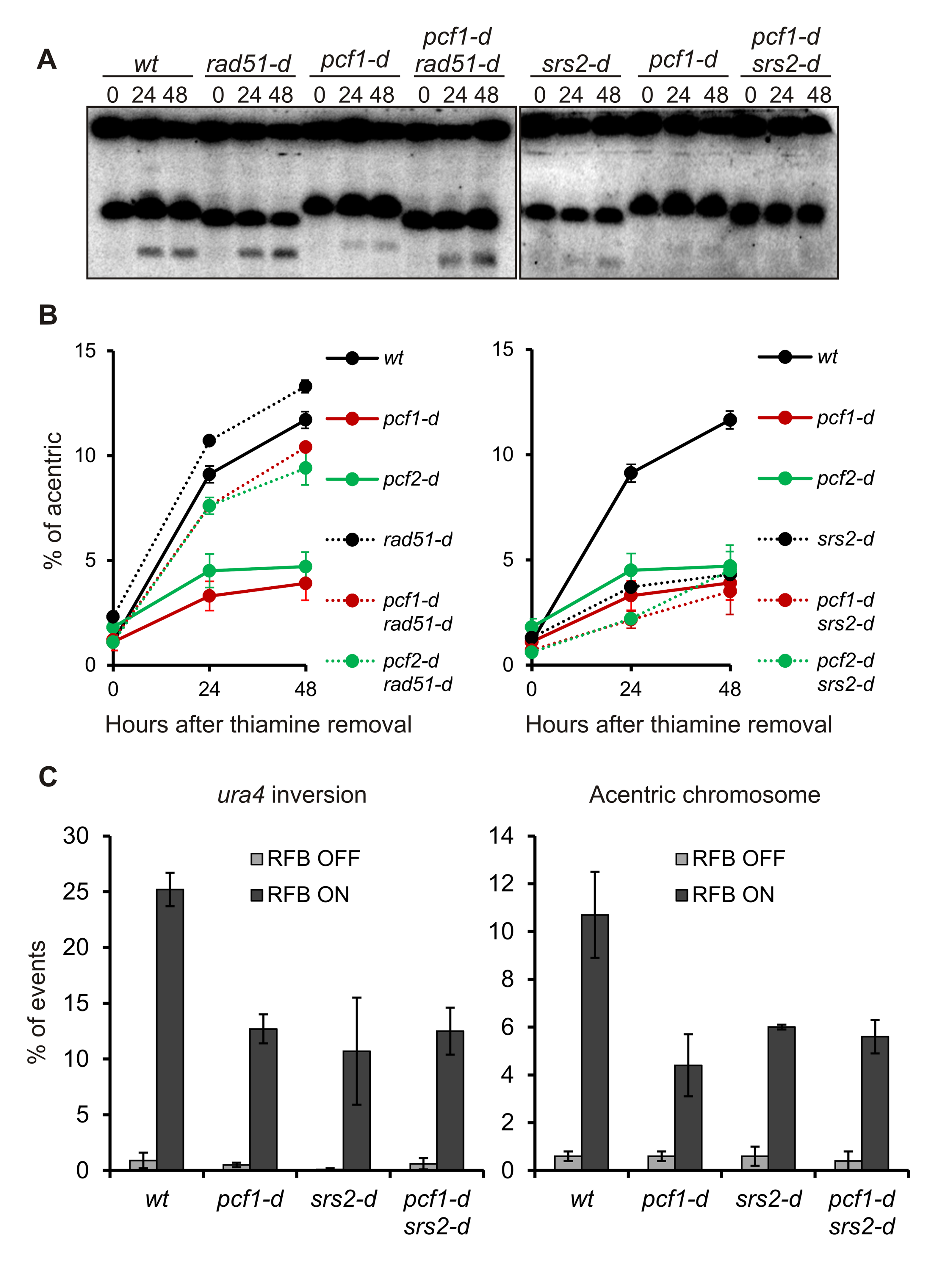

Supplement: Figure S4 — Genetic interactions between CAF-1–defective strains, rad51 and srs2 . (A) Chromosomes from indicated strains and conditions were separated by PFGE and analyzed by Southern blotting using rng3 probe, located tel proximal from ura4. Cells were grown with (RFB OFF, time 0) or without thiamine (RFB ON) for 24 and 48 h. (B) Quantification of the amounts of acentric chromosomes seen in panel A. Values correspond to the mean of at least three independent experiments ±SEM. Refer to Data S1, sheet 14. (C) Quantification of ura4 inversion and acentric chromosome in indicated strains and conditions (see Figure S2 for details). Values correspond to the mean of at least three independent experiments ±SD. Refer to Data S1, sheet 15. (TIF) [file pbio.1001968.s004.tif]

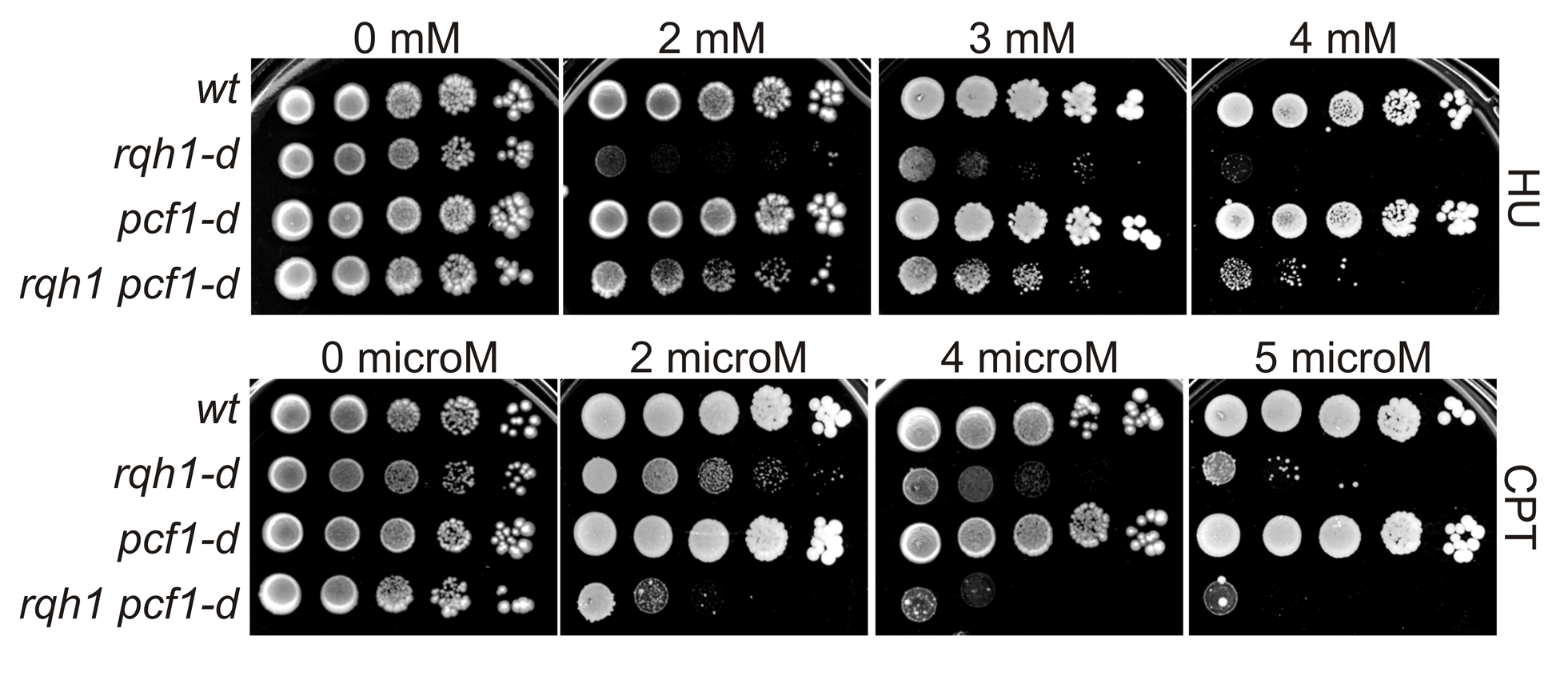

Supplement: Figure S5 — Genetic interactions between rqh1 and pcf1 in response to replication stress. Serial 10-fold dilution from indicated strains spotted onto media containing indicated hydroxyurea (HU, top panel) or camptothecin (CPT, bottom panel) concentration. (TIF) [file pbio.1001968.s005.tif]

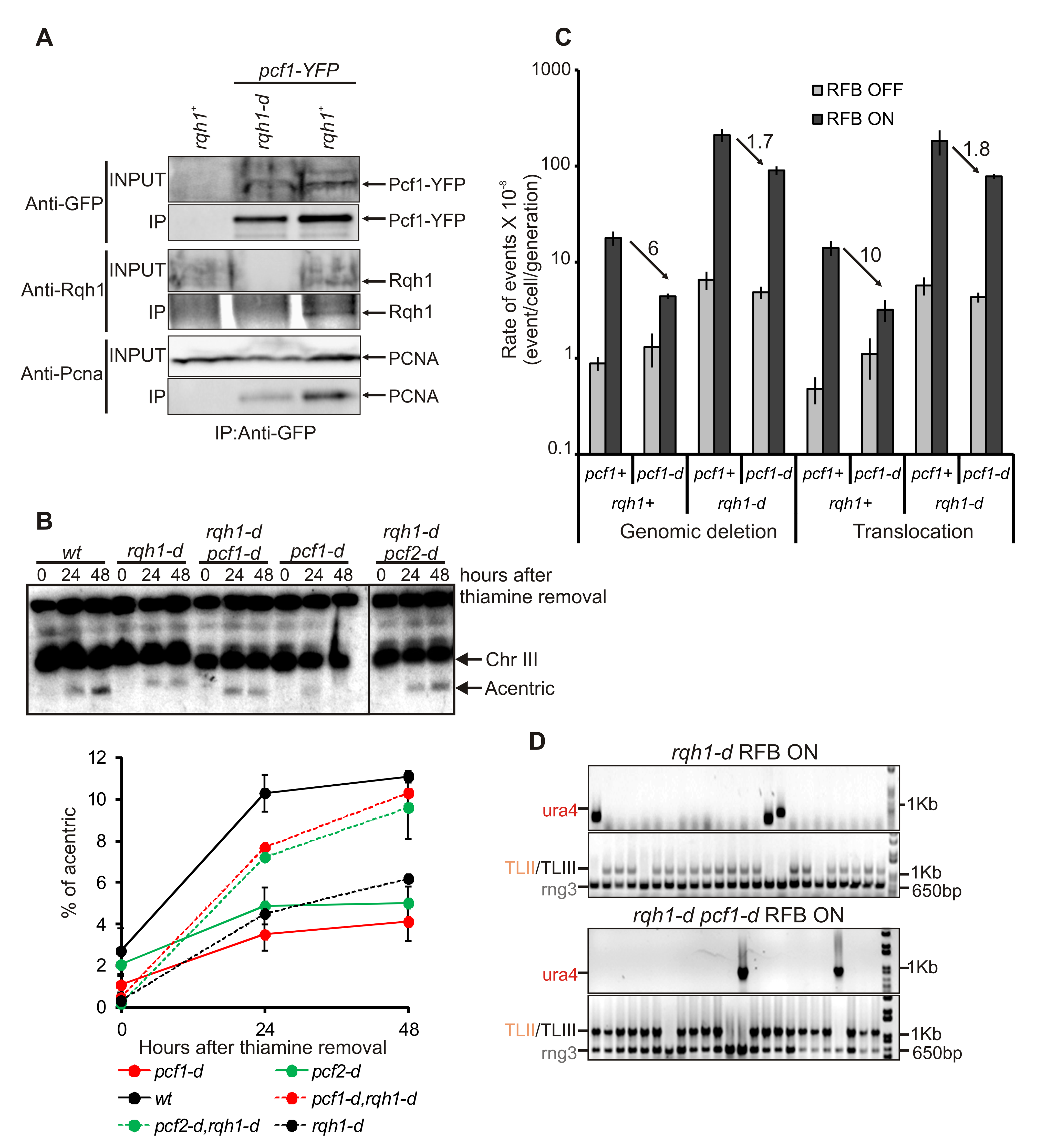

Supplement: Figure S6 — Functional and physical interactions between Pcf1, the large subunit of CAF-1, and Rqh1. (A) Immunoprecipitation of Pcf1-YFP by anti-GFP antibody in indicated strains. After protein separation by electrophoresis, samples were analyzed using anti-GFP antibody to reveal Pcf1-YFP, using Rqh1 antibody (a gift from J. Murray) to reveal endogenous Rqh1, and anti-PCNA. (B, Top panel) Chromosomes from indicated strains and conditions were separated by PFGE and analyzed by Southern blotting using Rng3 probe, located tel proximal from ura4. Cells were grown with (RFB OFF) or without thiamine (RFB ON) for 24 or 48 h. (Bottom panel) Quantification of the amounts of acentric chromosomes. Values correspond to the mean of at least three independent experiments ±SEM. Refer to Data S1, sheet 16. (C) Rate of genomic deletion and translocation for the strains indicated; ON and OFF refers to the RTS1-RFB being active or not, respectively. The percentage of deletion and translocation events, as determined by the PCR assay, was used to balance the rate of ura4 loss. The values reported are means of at least three independent median rates ±SD. Statistical significance was calculated using the nonparametric Mann–Whitney U test. Refer to Data S1, sheet 17. (D) Representative PCR amplification from 5-FOAR colonies from indicated strains and conditions. PCR products and their sizes are indicated on the figure. (TIF) [file pbio.1001968.s006.tif]

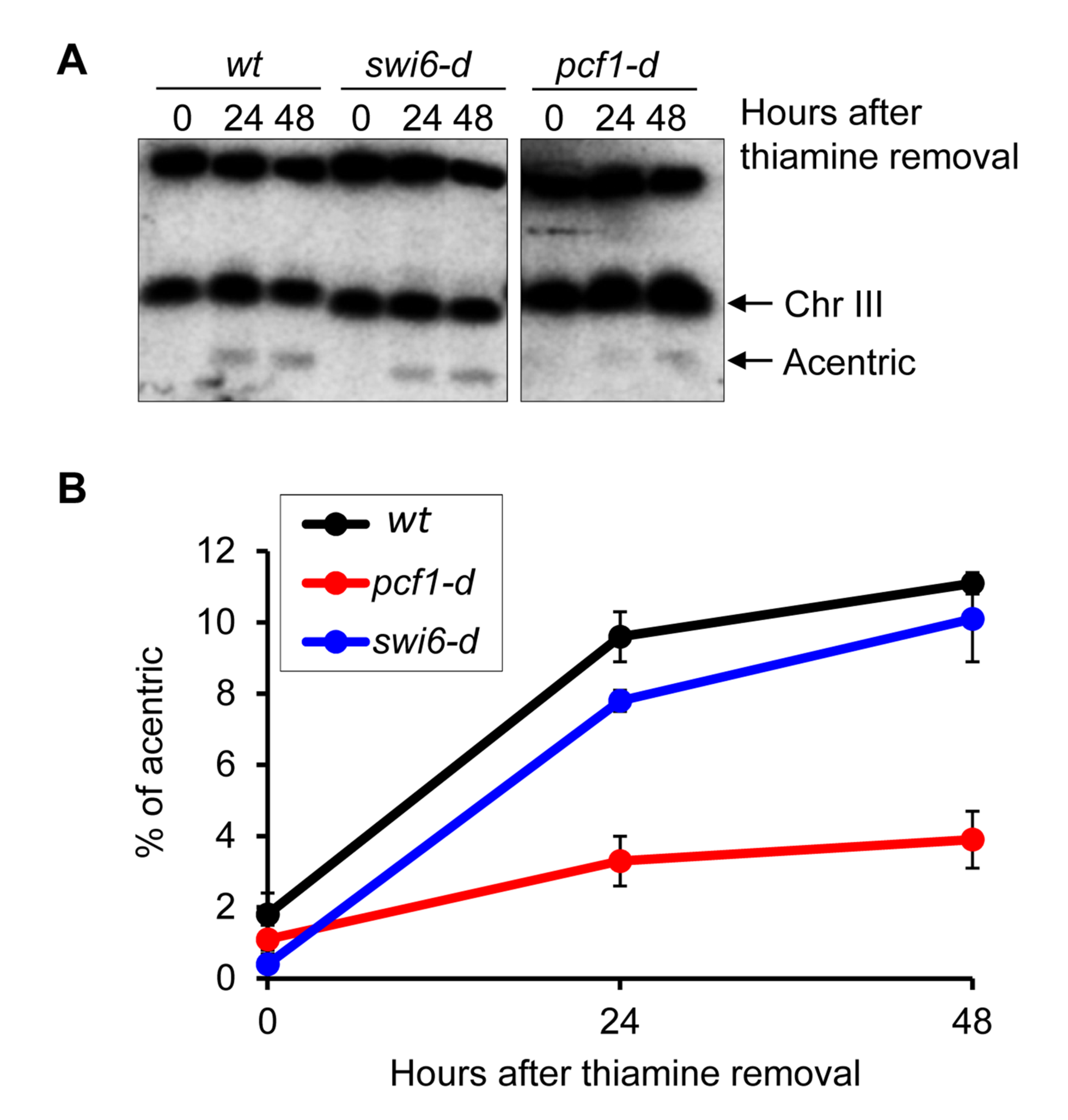

Supplement: Figure S7 — The heterochromatin factor Swi6 is not involved in template switch. (A) Chromosomes from indicated strains and conditions were separated by PFGE and analyzed by Southern blotting using Rng3 probe, located tel proximal from ura4. Cells were grown with (RFB OFF) or without thiamine (RFB ON) for 24 and 48 h. (B) Quantification of the amounts of acentric chromosomes seen in panel A. Values correspond to the mean of at least three independent experiments ±SEM. Refer to Data S1, sheet 18. (TIF) [file pbio.1001968.s007.tif]

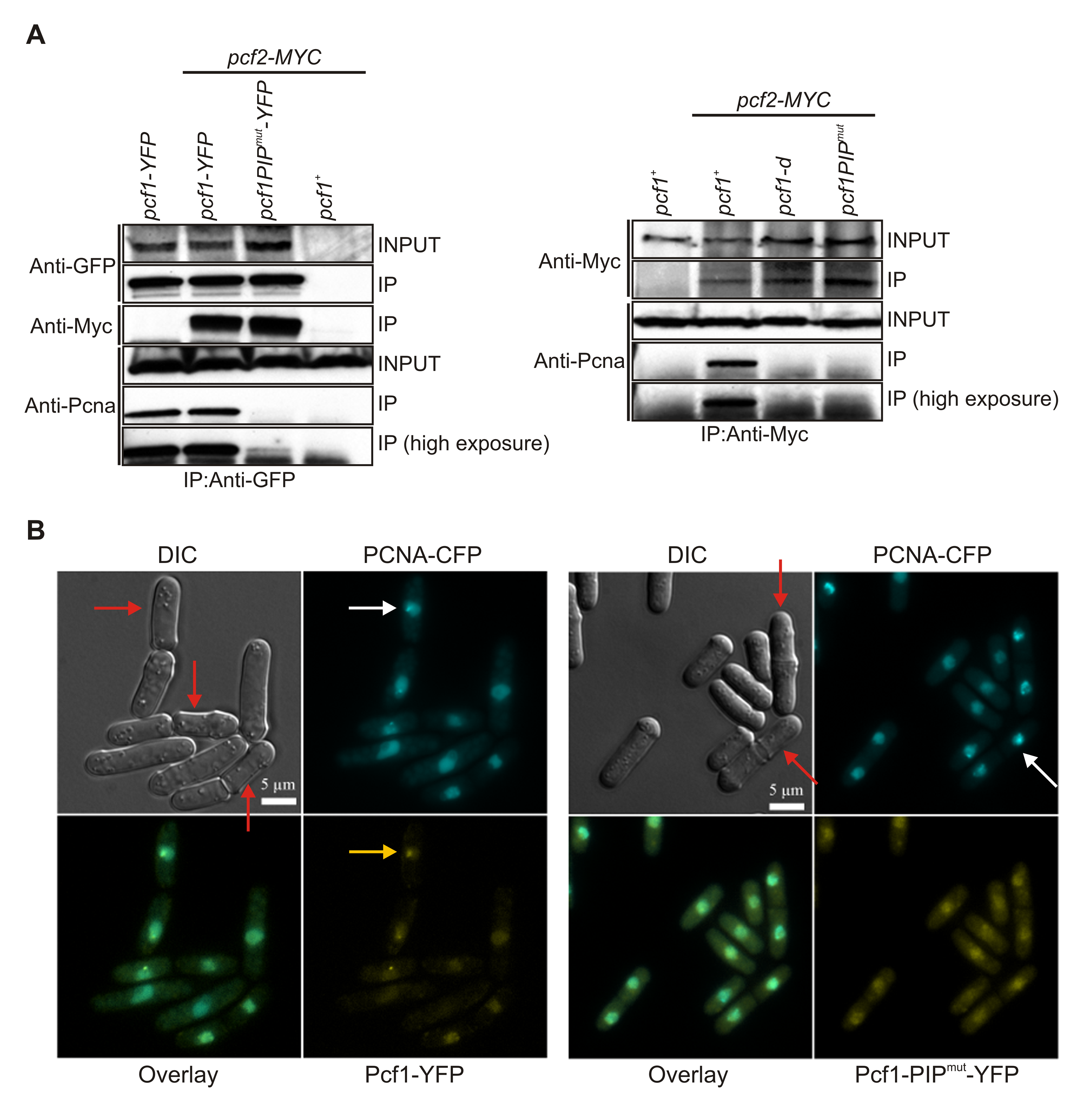

Supplement: Figure S8 — CAF-1 and PCNA interaction targets CAF-1 in replication foci. (A, Left panel) Immunoprecipitation of Pcf1-YFP by anti-GFP antibody in indicated strains. After protein separation by electrophoresis, samples were analyzed using anti-GFP antibody to reveal Pcf1-YFP, or Pcf1PIPmut-YFP, using anti-Myc to reveal Pcf2-MYC, and anti-PCNA. The mutation of the PIP box of Pcf1 severely impaired Pcf1/PCNA interaction without affecting Pcf1/Pcf2 interaction. (Right panel) Immunoprecipitation of Pcf2-MYC by anti-Myc antibody in indicated strains. After protein separation by electrophoresis, samples were analyzed using anti-Myc to reveal Pcf2-MYC, and anti-PCNA. The mutation of the PIP box of Pcf1 is sufficient to impair Pcf2/PCNA interaction. (B) Co-localization of PCNA-CFP and wt Pcf1-YFP (left panels) or Pcf1PIPmut-YFP (right panels). Differential interferential Contrast (DIC), CFP (Cyan), YFP (Yellow), and overlay acquisition signals are presented. The scale is indicated on the figure. Red arrows indicate S-phase cells (septated cells). White and yellow arrows indicate examples of S-phase nuclei containing PCNA or Pcf1 foci, respectively. (TIF) [file pbio.1001968.s008.tif]
