# Supplementary material for: The Chromatin Assembly Factor 1 Promotes Rad51-Dependent Template Switches at Replication Forks by Counteracting D-Loop Disassembly by the RecQ-Type Helicase Rqh1
Source: PLoS Biol. 2014 Oct 14;12(10):e1001968. doi: 10.1371/journal.pbio.1001968 (PMC4196752; doi:10.1371/journal.pbio.1001968)
Supplement: Table S1 — Strains used in this study. (DOCX) [file pbio.1001968.s009.docx]

**Table S1: Strains used in this study.**

| Strains | Genotype | Reference |
| --- | --- | --- |
| SL350 | *h^-^ sup35:nmt41:rtf1 ade6-704 leu1-32 t-ura4<ori* | Lambert 2005 |
| VP50 | *h^-^ sup35:nmt41:rtf1 pcf1::KanMX ade6-704 leu1-32 t-ura4<ori* | This study |
| VP52 | *h^-^ sup35:nmt41:rtf1 pcf2::KanMX ade6-704 leu1-32 t-ura4<ori* | This study |
| VP56 | *h^-^ sup35:nmt41:rtf1 pcf3::KanMX ade6-704 leu1-32 t-ura4<ori* | This study |
| YC5 | *h^-^ sup35:nmt41:rtf1 ade6-704 leu1-32 t-ura4sd20-ori* | Iraqui 2012 |
| YC13 | *h^-^ sup35:nmt41:rtf1 ade6-704 leu1-32 t-ura4sd20<ori* | Iraqui 2012 |
| YC184 | *h^-^ sup35:nmt41:rtf1 pcf1::KanMX ade6-704 leu1-32 t-ura4sd20-ori* | This study |
| YC188 | *h^-^ sup35:nmt41:rtf1 pcf1::KanMX ade6-704 leu1-32 t-ura4sd20<ori* | This study |
| SL337 | *h^-^ smt0 sup35:nmt41:rtf1 ade6-704 leu1-32 t>ura4<ori* | Lambert 2010 |
| VP15 | *h^-^ smt0 sup35:nmt41:rtf1 pcf1::KanMX ade6-704 leu1-32 t>ura4<ori* | This study |
| VP9 | *h^-^ smt0 sup35:nmt41:rtf1 pcf2::KanMX ade6-704 leu1-32 t>ura4<ori* | This study |
| VP16 | *h^-^ smt0 sup35:nmt41:rtf1 pcf3::KanMX ade6-704 leu1-32 t>ura4<ori* | This study |
| VP2 | *h^-^ smt0 sup35:nmt41:rtf1 rad52::KanMX ade6-704 leu1-32 t>ura4<ori* | Lambert 2010 |
| VP94 | *h^-^ smt0 sup35:nmt41:rtf1 pcf1::KanMX rad52::NatMX ade6-704*  *leu1-32 t>ura4<ori* | This study |
| VP120 | *h^-^ smt0 sup35:nmt41:rtf1 pcf1::KanMX pcf2::KanMX pcf3::KanMX*  *ade6-704 leu1-32 t>ura4<ori* | This study |
| VP307 | *h^-^ smt0 sup35:nmt41:rtf1 ade6-704 pcf1PIP^mut^ leu1-32 t>ura4<ori* | This study |
| SL511 | *h^-^ smt0 sup35:nmt41:rtf1 rqh1::KanMX ade6-704 leu1-32 t>ura4<ori* | Lambert 2010 |
| VP236 | *h^-^ smt0 sup35:nmt41:rtf1 rqh1::KanMX pcf1::KanMX ade6-704 leu1-32 t>ura4<ori* | This study |
| VP241 | *h^-^ smt0 sup35:nmt41:rtf1 rqh1::KanMX pcf2::KanMX ade6-704 leu1-32 t>ura4<ori* | This study |
| VP21 | *h^+^ sup35:nmt41:rtf1 rad22:GFP:KanMX ade6-704 leu1-32 t>ura4<ori* | Lambert 2005 |
| VP219 | *h^-^ smt0 sup35:nmt41:rtf1 rad22:GFP:KanMX pcf2::KanMX ade6-704 leu1-32 t>ura4<ori* | This study |
| VP229 | *h^-^ smt0 sup35:nmt41:rtf1 rad22:GFP:KanMX pcf1::KanMX ade6-704 leu1-32 t>ura4<ori* | This study |
| VP294 | *h^-^ smt0 srs2::NatMX rqh1::kanMX ade6-704 leu1-32 ura4-D18* | This study |
| VP300 | *h^-^ smt0 srs2::NatMX rqh1::kanMX pcf1::ura4^+^ ade6-704 leu1-32 ura4-D18* | This study |
| VP318 | *h^-^ smt0 srs2::NatMX rqh1::kanMX pcf2::ura4^+^ ade6-704 leu1-32 ura4-D18* | This study |
| SL965 | *h^-^ smt0 srs2::ura4^+^ rqh1::kanMX rad51::arg3^+^ ura4-D18 his3-D1 arg3-D4* | Doe 2004 (MCW1097) |
| VP69 | *h^-^ smt0 sup35:nmt41:rtf1 swi6::NatMX ade6-704 leu1-32 t>ura4<ori* | This study |
| SL 917 | *h^-^ smt0, ura4-D18 ade6-375 int:puc8/ura4+/ade6-469* | Hartsuiker 2001 |
| VP152 | *h^-^ smt0, pcf1::Kan ura4-D18, ade6-375 int:puc8/ura4+/ade6-469* | This study |
| VP155 | *h^-^ smt0, pcf2::Kan ura4-D18 ade6-375 int:puc8/ura4+/ade64-69* | This study |
| VP158 | *h^-^ smt0, pcf3::Kan ura4-D18 ade6-375 int:puc8/ura4+/ade64-69* | This study |
| VP186 | *h^-^ smt0/ h^-^ smt0 ade6 M-210/ade6 M-216 ura4-D18/ura4^+^* | Hartsuiker 2001 |
| VP190 | *h^-^ smt0/ h^-^ smt0 ade6 M-210/ade6 M-216 ura4-D18/ura4^+^ ade6 M-216 pcf1::kanMX/pcf1::KanMX* | This study |
| VP198 | *h^-^ smt0/ h^-^ smt0 ade6 M-210/ade6 M-216 ura4-D18/ura4^+^ ade6 M-216 pcf2::kanMX/pcf2::KanMX* | This study |
| VP203 | *h^-^ smt0/ h^-^ smt0 ade6 M-210/ade6 M-216 ura4-D18/ura4^+^ ade6 M-216 pcf3::kanMX/pcf3::KanMX* | This study |
| VP393 | *h^-^ smto, pcf1:YFP:KanMX ade6-704 leu 1-32 ura4-D18* | This study |
| VP425 | *h^-^ smto, pcf2:Myc:KanMX pcf1PIP^mut^:YFP:KanMX ade6-704 leu 1-32 ura4-D18* | This study |
| VP426 | *h^-^ smto, pcf2:Myc:KanMX pcf1:YFP:KanMX ade6-704 leu1-32 ura4-D18* | This study |
| VP409 | *h^-^ smto, pcf2:Myc:KanMX ade6-704 leu1-32 ura4-D18* | This study |
| VP434 | *h^-^ smto, pcf2:Myc:KanMX pcf1::ura4^+^ ade6-704 leu1-32 ura4-D18* | This study |
| VP430 | *h^-^ smto, pcf2:Myc:KanMX pcf1PIP^mut^ ade6-704 leu1-32 ura4-D18* | This study |
| VP217 | *h^-^ pcf1:YFP:KanMX ura4:pECFP:pcn1 pcn1^+^* | This study |
| VP440 | *h^-^ smto pcf1PIP^mut^:YFP:KanMX ura4::pECFP:pcn1 pcn1^+^* | This study |
| SL530 | *h^-^ smt0 sup35:nmt41:rtf1 mus81::KanMX ade6-704 leu1-32 t>ura4<ori* | Lambert 2010 |
| VP533 | *h^-^ smt0 sup35:nmt41:rtf1 mus81::KanMX pcf1::KanMX ade6-704 leu1-32 t>ura4<ori* | This study |
| SL279 | *h^-^ smt0 sup35:nmt41:rtf1 rad51::KanMX ade6-704 leu1-32 t>ura4<ori* | Lambert 2005 |
| VP121 | *h^+^ sup35:nmt41:rtf1 rad51::KanMX pcf1::KanMX ade6-704 leu1-32 t>ura4<ori* | This study |
| VP133 | *h^-^ smt0 sup35:nmt41:rtf1 rad51::KanMX pcf2::KanMX ade6-704 leu1-32 t>ura4<ori* | This study |
| SL748 | *h^-^ sup35:nmt41:rtf1 srs2::NATMX ade6-704 leu1-32 t>ura4<ori* | Lambert 2010 |
| VP089 | *h^-^ smt0 sup35:nmt41:rtf1 srs2::NATMX pcf1::KanMX ade6-704 leu1-32 t>ura4<ori* | This study |
| VP098 | *h^-^ smt0 sup35:nmt41:rtf1 srs2::NATMX pcf2::KanMX ade6-704 leu1-32 t>ura4<ori* | This study |
| VP518 | *h^-^ smt0 pcf1:YFP:KanMX rqh1::kanMX ade6-704 leu1-32 ura4-D18* | This study |
| VP520 | *h^-^ smt0 rqh1:Myc:KanMX pcf1:YFP:KanMX ade6-704 leu1-32 ura4-D18* | This study |
| SL782 | *h^+^ rqh1:Myc:KanMX ade6-704 leu1-32 ura4-D18* | Gift from J. Murray |
| SL633 | *h^-^ rev1::kanMX ade6-704 leu1-32 ura4-D18* | This study |
| JH7 | *h^-^ pcf1::ura4 rev1::kanMX ade6-704 leu1-32 ura4-D18* | This study |
| II1 | *h^-^ smt0 rad8::HYGMX ade6-704 leu1-32 ura4-D18* | This study |
| JH3 | *h^-^ smt0 rad8::HYGMX pcf1::ura4 ade6-704 leu1-32 ura4-D18* | This study |
| VP285 | *h^+^ pcf1::ura4 ade6-704 leu1-32 ura4-D18* | This study |
| VP316 | *h^-^ smt0 rqh1::KanMX pcf1::ura4 ade6-704 leu1-32 ura4-D18* | This study |
| SL134 | *h^+^ rad51::KanMX ade6-704 leu1-32 ura4-D18* | This study |
