# Supplementary material for: The Chromatin Assembly Factor 1 Promotes Rad51-Dependent Template Switches at Replication Forks by Counteracting D-Loop Disassembly by the RecQ-Type Helicase Rqh1
Source: PLoS Biol. 2014 Oct 14;12(10):e1001968. doi: 10.1371/journal.pbio.1001968 (PMC4196752; doi:10.1371/journal.pbio.1001968)
Supplement: Table S2 — Primers used for ChIP/qPCR. (DOCX) [file pbio.1001968.s010.docx]

**Table S2: Primers used for ChIP/qPCR**

| **Primer name** | **location** | **sequence** |
| --- | --- | --- |
| R3F | Ura4-3 | TTCTGTTCCAACACCAATGTTT |
| R3R | Ura4-3 | TGTACAAAGCCAATGAAAGATG |
| ura4F1 | Ura4-2 | GACTCCACGACCAACAATGA |
| ura4R1 | Ura4-2 | CTGGTATCGGCTTGGATGTT |
| uraR2 | Ura4-1 | CAAATTCGCAGACATTGGAA |
| uraF2 | Ura4-1 | TGATATGAGCCCAAGAAGCA |
| L5F | *RTS1*-RFB | AGGGCATTAAGGCTTATTTACAGA |
| L5R | *RTS1*-RFB | TCACGTTTAATTTCAAACATCCA |
| L3F | *RTS1*-RFB | TTTAAATCAAATCTTCCATGCG |
| L3R | *RTS1*-RFB | TGTACCCATGAGCAAACTGC |
| L400F | 0.4 Kb away from *RTS1*-RFB | ATCTGACATGGCATTCCTCA |
| L400R | 0.4 Kb away from *RTS1*-RFB | GATGCCAGACCGTAATGACA |
| L600F | 0.6 Kb away from *RTS1*-RFB | CCATTGACTAGGAGGACTTTGAG |
| L600R | 0.6 Kb away from *RTS1*-RFB | CCCTGGCGGTTGTAGTTAGT |
| L900F | 0.9 Kb away from *RTS1*-RFB | AACGGTTGTAGAAGACGAGCA |
| L900R | 0.9 Kb away from *RTS1*-RFB | TGTAAGCACACCTTCAATGTATCA |
| L1400F | 1.4 Kb away from *RTS1*-RFB | AACATCGGTGACCTCGTTCT |
| L1400R | 1.4 Kb away from *RTS1*-RFB | CTCTTCGCTCCAAGCGTTAT |
| L1800F | 1.8 Kb away from *RTS1*-RFB | GATGCCAGACCGTAATGACA |
| L1800R | 1.8 Kb away from *RTS1*-RFB | TGAATACGCCGTTACTCCTAAAG |
| L2200F | 2.2 Kb away from *RTS1*-RFB | AAGGCAAGAAACGCTGAGAC |
| L2200R | 2.2 Kb away from *RTS1*-RFB | GGCATGCATACTACCCGATAA |
| L2600F | 2.6 Kb away from *RTS1*-RFB | ACAGATAAATGGCGCAAACC |
| L2600R | 2.6 Kb away from *RTS1*-RFB | CGAACACAAGTGTGGTCTTTG |
| Ade6-23 | Control | GGCTGCCTCTACCATCATTC |
| Ade6-25 | Control | TTAAGCTGAGCTGCCAAGGT |
